# Characterization and quantification of the phytochemical constituents and anti-inflammatory properties of *Lindera aggregata*†

**DOI:** 10.1039/d4ra05643d

**Published:** 2024-11-11

**Authors:** Song-Song Wen, Yong-Jun Liu, Yu-Lin Liu, Yan Zhao, Xiao-Xi Xu, Chang-Chuan Guo, Chong Niu, Wei-Jian Wang, Yu-Wen Xu, Na Zhang

**Affiliations:** a Department of Pharmaceutics, Key Laboratory of Chemical Biology (Ministry of Education), School of Pharmaceutical Sciences, Cheeloo College of Medicine, Shandong University Jinan 250012 China zhangnancy9@sdu.edu.cn; b NMPA Key Laboratory for Research and Evaluation of Generic Drugs, Shandong Research Center of Engineering and Technology for Consistency Evaluation of Generic Drugs, Shandong Institute for Food and Drug Control Jinan 250101 China 13553158409@163.com; c Department of Marine Pharmacology, College of Food Science and Technology, Shanghai Ocean University Shanghai 201306 China; d Beijing Normal University Beijing 100875 China

## Abstract

The dry roots of *Lindera aggregata* (Sims) Kosterm have a long-standing history in traditional Chinese medicine, renowned for their ability to regulate vital energy, relieve pain, warm the kidney, and dissipate cold. Recently, *L. aggregata* has been approved as a new food resource. To gain insights into the bioactive phytochemicals in *L. aggregata*, an ultrahigh-performance liquid chromatography coupled with high-resolution electrospray ionization quadrupole orbitrap spectrometry method was developed to investigate the chemical profiles of the ethanol extract of *L. aggregata*. This approach identified 80 compounds, predominantly alkaloids and sesquiterpenoids. Furthermore, 16 selected compounds were simultaneously quantified using the parallel reaction monitoring mode. The quantification method was validated and showed good linearity, sensitivity, and accuracy. The anti-inflammatory activities of the ethanol extract and selected compounds were assessed *in vitro* using lipopolysaccharide-stimulated RAW 264.7 macrophages. The results revealed that the ethanol extract of *L. aggregata* and norisoboldine, isolinderalactone, methyllinderone, and linderin B inhibited the production or expression of nitric oxide, inducible nitric oxide synthase (iNOS), tumor necrosis factor-α, and interleukin-6. Molecular docking of iNOS with isolinderalactone, methyllinderone, and linderin B showed that hydrogen bonds, π–π interactions, and hydrophobic interactions contributed to their iNOS inhibitory effects. The results offer insights that may be instrumental in enhancing the quality control for *L. aggregata.*

## Abstract 2

This study developed a method to analyze the bioactive compounds in *Lindera aggregata* roots, identifying 80 compounds, with 16 quantified, and found that specific compounds exhibit anti-inflammatory effects.

## Graphical abstract

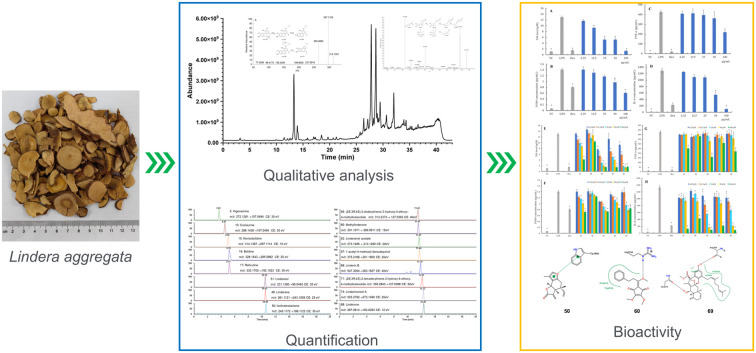

## 1. Introduction

Medicaments used in traditional medicine (TM) are predominantly derived from natural sources. In TM, the equivalent of “clinical trials” has been practiced since ancient times. Specifically, in Traditional Chinese Medicine (TCM), extensive experience and advancements have been accumulated and refined over millennia. These include methods of preparation, herb selection, identification of medicinal materials, and determining the optimal time for harvesting various plants. The dry roots of *Lindera aggregata*, which belong to the Lauaceae family and are known as Radix *Linderae*, hold a significant place in traditional Chinese medicine for treating numerous ailments over a long historical span. In line with traditional Chinese medicine principles, *L. aggregata*, commonly referred to “Wuyao”, is believed to invigorate and harmonize bodily metabolism, offering effects that promote Qi, alleviate pain, warm the kidney, and dispel coldness.^1^ Characterized by its pungent flavor, mild aroma, and association with the kidney and stomach meridians, *L. aggregata* has been used to enhance kidney functionality. Recognized for its substantial medicinal value and wide-ranging pharmacological effects, *L. aggregata* has garnered increasing attention in recent years. Over the past few decades, researchers have explored *L. aggregata* from diverse perspectives, encompassing the profiling of phytochemical constituents, understanding pharmacological mechanisms, and establishing methods for quality control.^2^ So far, more than 250 compounds, including flavonoids, alkaloids, terpenes, volatile compounds, and tannins, have been isolated and identified from *L. aggregata*.^2–7^ Ongoing pharmacological studies have demonstrated its potential in various areas, such as anti-cancer, anti-inflammatory, anti-arthritis, anti-bacterial, anti-oxidation, anti-diabetic nephropathy, hepatoprotective, and lipid-lowering effects.^2^ These pharmacological properties have been extensively investigated, with a focus on the crude extract of the root tuber, leaf extract, as well as the chemical compounds.

Recognized both as a medicinal herb and a food-related plant, *L. aggregata* gained approval as a new food resource in China in 2012, signifying its vast development prospects. Consequently, the herb market has witnessed a significant surge in demand for *L. aggregata*. Its cultivation and utilization in the realms of food and healthcare harbor immense potential for growth and profitability. The growing enthusiasm for the supply of *L. aggregata* necessitates comprehensive analytical characterization of its bioactive constituents. This approach aims to comprehensively understand their collective impact on both food properties and human health.

While numerous monomeric compounds from *L. aggregata* have been successfully isolated and identified, research investigations have predominantly focused on the pharmacological effects of the crude extract or primary chemical components. Consequently, there is limited information regarding quality control research, especially concerning the swift identification and quantification of active constituents.^7,8^ To date, only a few representative sesquiterpenoids and alkaloids have been quantified using LC-MS method. Furthermore, only two active constituents, linderane and norisoboldine, have been designated as the chemical markers for quality control of *L. aggregata* in China Pharmacopeia 2020. However, it is widely acknowledged that the therapeutic efficacy of *L. aggregata* relies on the intricate interactions among numerous ingredients in combination, which are different from typical pharmaceutical chemicals. Relying on the determination of only two compounds may not adequately represent the overall clinical therapeutic effects. Therefore, there is a pressing need for a rapid and reliable method to comprehensively determine the chemical profiles of *L. aggregata*.

Ultra-high performance liquid chromatography (UHPLC)-MS/MS, renowned for its superior speed, enhanced sensitivity, and specificity compared to HPLC-UV analysis, has gained increased attention in the analysis of traditional Chinese medicines. Hence, the objective of this study is to establish a rapid, sensitive, and efficient ultrahigh-performance liquid chromatography coupled with high-resolution electrospray ionization quadrupole orbitrap mass spectrometry (UHPLC-HR-ESI-Q-Orbitrap) method for the qualitative analysis, followed by parallel reaction monitoring (PRM) mode, for the simultaneous quantification of phytochemicals in *L. aggregata*. The anti-inflammatory effects of the ethanol extract of *L. aggregata* and selected compounds were measured as the ability to suppress nitril oxide (NO), tumor necrosis factor α (TNF-α), interleukin (IL)-6, and nitric oxide synthase (iNOS) expression in lipopolysaccharide (LPS)-stimulated RAW 264.7 mouse macrophages. In addition, a molecular docking method was carried out to elucidate the protein–ligand interactions between iNOS and the potential bioactive compounds.

## 2. Materials and methods

### 2.1. Reagents, chemicals, and plant materials

Ethanol used for extraction was analytical grade, and purchased from Fisher Scientific (Waltham, MA, USA). LC/MS-grade acetonitrile, methanol, water, and trifluoroacetic acid purchased from Merck (Darmstadt, Germany) were used in the sample preparation and UHPLC-MS analysis. LC-MS-grade formic acid was obtained from Fisher Scientific Co. (Waltham, MA, USA). Norisoboldine (P/N, 111 825-201802) and linderane (P/N, 111 568-201906) were purchased from the National Institute of Food and Drug Control (Beijing, China). Boldine (P/N, X23N9Y73113), isolinderalactone (P/N, S30HB196732), reticuline (P/N, N28HB202502), linderone (P/N, D0HB03066), and methyllinderone (P/N, D0HB03065) were purchase from Shanghai Yuanye Bio-Technology Co., Ltd (Shanghai, China). Higenamine (P/N, MUST-22032110), lindenenol (P/N, MUST-22032904), and linderene acetate (P/N, MUST-22111917) were purchased from Chengdu MUST Biotechnology Co., Ltd (Chengdu, China). Coclaruine (P/N, 220 511) was obtained from Chengdu Herb Substance Company (Chengdu, China). The standards of linderin B, lindechunisin A, 1-acetyl-4-methoxyl-denudaquinol, (2*E*,3*R*,4*S*)-2-tetradecylinene-3-hydroxy-4-ethoxy-4-methylbutanolide, and (2*E*,3*R*,4*S*)-2-dodecylinene-3-hydroxy-4-ethoxy-4-methylbutanolide were obtained from our laboratory. Their structures were unambiguously characterized by ^1^H NMR, ^13^C NMR, 2D NMR and HR-ESIMS techniques.

Dulbecco's modified Eagle medium (DMEM), penicillin and streptomycin were purchased from Gibco BRL (Grand Island, NY, USA); new-born calf serum (NBCS) was purchased from PAA Laboratories GmbH, Austria; 3-[4,5-dimetylthiazol-2-yl]-2,5-diphenyl-tetrazolium bromide (MTT), Tween 20, bovine serum albumin (BSA), sodium dodecyl sulfate (SDS), dithiotheitol (DTT), phenylmethylsulfonyl fluoride (PMSF), and LPS were purchased from Sigma Chemical Co. (St. Louis, MO, USA). TNF-α, IL-6, and nitric oxide detection kits were purchased from Nanjing Jiancheng Bioengineering Institute (Nanjing, China).

The dried roots of *L. aggregata* were purchased from Bozhou Hu Herb Company, China, in March 2023, and were authenticated by Dr Qiyan Li, Shandong Institute for Food and Drug Control. The voucher specimen was deposited at the Shandong Institute for Food and Drug Control, Jinan, Shandong Province, China.

### 2.2. Extraction and sample preparation

The dried roots of *L. aggregata* underwent comminution using a mill to pass through a 40-mesh sieve. The L.*aggregata* root powder (150.0 g) was extracted with 80% ethanol (500 mL) under reflux for three times (2 h for each time). The ethanol extract was filtered and combined, and the solvent was evaporated under vacuum to obtain a crude extract. The crude extract was successively lyophilized for subsequent analysis. The extraction yield was 13.6 g of crude extract from 150 g raw herb material. The solution of the freeze-dried *L. aggregata* ethanolic extract (LAE, 0.5 mg mL^−1^) was prepared in methanol under sonication, and filtered through a 0.22 μm polyvinylidene difluoride membrane prior to LC-MS detection.

### 2.3. Preparation of standard stock solutions

Based on the compound identification results, individual stock solutions (1.0 mg mL^−1^) of higenamine, coclaruine, norisoboldine, boldine, reticuline, linderane, isolinderalactone, lindenenol, linderene acetate, linderone, methyllinderone, linderin B, lindechunisin A, 1-acetyl-4-methoxyl-denudaquinol, (2*E*,3*R*,4*S*)-2-tetradecylinene-3-hydroxy-4-ethoxy-4-methylbutanolide, and (2*E*,3*R*,4*S*)-2-dodecylinene-3-hydroxy-4-ethoxy-4-methylbutanolide were individually prepared in LC-MS-grade methanol. Subsequently, stock solutions containing a mixture of these 16 analytes were prepared and further diluted in the appropriate concentration using methanol to yield a series of concentrations from 1.0 ng mL^−1^ to 1500 ng mL^−1^. All the prepared stock solutions were stored in the refrigerator at −20 °C until subsequent analysis.

### 2.4. UHPLC-MS/MS analysis for qualitative study

The quantitative analysis was performed using a Vanquish Flex Binary UHPLC (Thermo Fisher Scientific, Waltham, MA, USA) with a Waters Acquity CSH C18 column (150 × 2.1 mm, 1.7 μm). The column temperature was maintained at 40 °C. The mobile phase A consisted of water and 0.1% formic acid. Mobile phase B comprised methanol and 0.1% formic acid. The flow rate was 0.5 mL min^−1^. The gradient elution conditions were as follows: 20% B (0–2 min); linear gradient from 20% B to 60% B (2–20 min); 60% B to 80% B (20–21 min); 80% B to 100% B (21–31 min); 100% B for 5 min (31–36 min); back to 20% B at 37 min; 20% B for 6 min balance (37–43 min). The injection volume was 2.00 μL.

The detection was carried out using a Q Exactive Plus mass spectrometer system (Thermo Fisher Scientific, Waltham, MA, USA). The parameters for the HRESI source were set as follows: capillary temperature at 275 °C; heater temperature at 300 °C; sheath gas flow, 50 arb; auxiliary gas flow, 10 arb; purge gas flow, 0 arb; spray voltage, 3.5 kV; S-lens RF level, 55%. The mass spectrometer adopted the Full-MS/ddMS2 scan in positive mode. Mass spectra were acquired in the range of 100 to 1200 *m*/*z*, and the resolution was set to 70 000. The automatic gain control (AGC) was 3 × 10^6^ and the injection time (IT) was 100 ms. For the MS/MS scan, the step-normalized collision energy was set to 20, 40, and 60 N with a resolution of 17 500. AGC is 1 × 10^5^ and IT is 50 ms. A data-dependent analysis scan was applied to trigger the second stage fragmentation, whereby the 20 most intense precursor ions at each scan point of the MS were selected as target precursor ions for subsequent MS/MS fragmentation.^9^

The raw data files obtained from UHPLC-Q-Orbitrap HRESI-MS analysis were processed using the Compound Discoverer 3.3 software (Thermo Fisher Scientific Inc. Waltham, USA). A chromatographic signal/noise (S/N) threshold of 3, mass tolerance of 5 ppm, and a minimum peak intensity of 2 × 10^3^ were used for compound detection. Compound identification was conducted by comparing the accurate mass, MS/MS fragmentation patterns, MzCloud, online metabolite databases of ChemSpider, the in-house compound library, and authentic standards. The in-house compound library on *L. aggregata* was established based on the reported literature. Approximately 600 compounds were collected from SciFinder and converted to individual structure files (.mol), forming the basis for our in-house library. Compound Discoverer 3.3 utilized exact mass, isotope pattern matching, as well as the MS and MS^2^ spectra, to conduct the structural identification. The compound database search parameters were adjusted according to the manufacturer's instructions. The collision energy tolerance was set at ±20%, with a match factor threshold of 75% and a maximum of 5 matching results for each compound. The best ion and related fragmentation data (highest resolution and intensity) of each compound were used to predict the elemental composition. Full-MS scans or predicted formulas, when available, were compared with the ChemSpider and in-house library. Fragmentation data (MS^2^) or predicted formulas, when available, were compared with the MzCloud database.

### 2.5. UHPLC-MS/MS quantification analysis

Sixteen selected compounds, including higenamine, coclaruine, norisoboldine, boldine, reticuline, linderane, isolinderalactone, lindenenol, linderene acetate, linderone, methyllinderone, linderin B, lindechunisin A, 1-acetyl-4-methoxyl-denudaquinol, (2*E*,3*R*,4*S*)-2-tetradecylinene-3-hydroxy-4-ethoxy-4-methylbutanolide, and (2*E*,3*R*,4*S*)-2-dodecylinene-3-hydroxy-4-ethoxy-4-methylbutanolide for quantitative study, were accomplished in parallel reaction mode (PRM). Chromatographic separation was performed on a Vanquish Flex Binary UHPLC (Thermo Fisher Scientific, Waltham, MA) with an ACE® Excel® C18-PFP column (2.1 × 100 mm, 3 μm, ACE, UK). The column temperature was set at 40 °C. The mobile phases consisted of 0.1% formic acid aqueous solution (A) and 0.1% formic acid dissolved in methanol (B), and the gradient elution program was as follows: 0–2 min, 40% B; 2–5 min, 40–100% B; 5–16 min, 100% B; 16–17 min, 100–40% B; 17–20 min, 40% B. The flow rate was maintained at 0.5 mL min^−1^, and the injection volume was 2 μL. The Q-orbitrap mass spectrometer was operated in positive mode. The settings used in HRESI were as follows: spray voltage, 3.5 kV; ion transfer tube temperature, 350 °C; vaporizer temperature, 400 °C sheath gas flow rate, 60 arb; auxiliary gas flow rate, 20 arb. Precursor ion scan mode was used for screening and PRM acquisition mode for quantification of the 16 compounds in LAE. Optimization of the MS/MS conditions for each compound was accomplished using standards through flow injection analysis.

### 2.6. Quantification method validation

The developed quantification method underwent validation in accordance with the International Conferences on Harmonization (ICH, Q2R1) guidelines, encompassing assessments for linearity, limits of detection (LOD), limits of quantification (LOQ), precisions, and recovery studies.^10^ Linearity was evaluated by constructing the calibration curves, correlating the peak areas against the nominal concentrations of calibration standards using weighted least-square linear regression. Each reference compound was tested at a minimum of five different concentrations to establish the correlation coefficient (*r*), slope, and intercept. The LOD and LOQ were defined as a S/N equal to 3 and 10, respectively. Precision and reproducibility were assessed by calculating the relative standard deviation (RSD) of the peak areas acquired from six replicates at a medium standard concentration. Accuracy was determined by measuring the mean recovery after adding the standard to actual samples at a medium spiked concentration with six replicates.

### 2.7. Anti-inflammatory activity assay

#### 2.7.1. Cell culture

RAW 264.7 cells, a mouse macrophage cell line, were obtained from American Type Culture Collection (ATCC No. TIB-71, Manassas, VA, USA). The cells were cultured in Dulbecco's modified Eagle's medium (DMEM) supplemented with 10% new-born calf serum, 100 units per mL penicillin, and 100 μg mL^−1^ streptomycin. Cultures were maintained at 37 °C in humidified air with 5% CO_2_. Dexamethasone (Dex) was used as a positive control in this experiment.

#### 2.7.2. Cell viability assay

Cell viability was examined using the MTT assay. RAW 264.7 cells were seeded at a density of 1 × 10^5^ cells per well in 96-well plates, and incubated overnight at 37 °C in a 5% CO_2_ environment. Following incubation, the cells were exposed to various concentrations of LAE (6.25, 12.5, 25, 50, and 100 μg mL^−1^) and compounds (6.25, 12.5, 25, 50, and 100 μM), both in the absence and presence of LPS (1 μg mL^−1^). Subsequently, 20 μL of MTT solution was added into each well, and the cells were incubated for 4 h at 37 °C. Afterward, the supernatant was removed, and formazan crystals were dissolved by adding 150 μL of DMSO to each well. The optical absorbance was measured at 540 nm using a plate reader.

#### 2.7.3. NO and iNOS protein assay

Nitrite (NO_2_^−^) levels in the culture medium were measured as an indicator of NO production using the Griess reaction, as described previously. Briefly, RAW 264.7 cells were seeded in a 6-well plate (1 × 10^6^ cells per well) and incubated for 24 h at 37 °C in 5% CO_2_. Plated cells were pretreated with the same concentrations in the cell viability assay for 2 h, and then stimulated with 1 μg mL^−1^ of LPS for an additional 22 h. The culture supernatant (50 μL) was mixed with the Griess reagent and incubated for 10 min. The absorbance of the mixture was measured at 540 nm using a microplate reader (Agilent BioTek Epoch). The amount of nitrite in the test samples was calculated using sodium nitrite standard curve. After the same above-described treatment, cells were lysed with RIPA (radioimmunoprecipitation assay) buffer [50 mM Tris-Cl (pH 8.0), 5 mM EDTA, 150 mM NaCl, 1% NP-40, 0.1% SDS and 1 mM phenylmethylsulfonyl fluoride]. Lysates were centrifuged at 12 000 rpm for 20 min. Supernatants were collected, and iNOS protein concentration was determined using a mouse iNOS ELISA kit (Abcam, Cambridge, USA). Dex (25 μM) was used as the positive control.

#### 2.7.4. Measurement of cytokines

The secretion of pro-inflammatory cytokines, including TNF-α and IL-6, was measured using an ELISA assay kit. RAW 264.7 cells were seeded in a 6-well plate (1 × 10^6^ cells per well) for 2 h and then incubated with various concentrations of LAE, compounds and LPS (1 μg mL^−1^) for 24 h. Subsequently, culture supernatants were collected, and the cytokines levels were quantified following the manufacturer's instructions. Absorbance was measured using a microplate spectrophotometer.

### 2.8. Molecular docking

The chemical structures of compounds with anti-inflammatory activities were selected as ligands for further molecular docking investigation. Ligands were prepared (minimization of energy done, hydrogen atoms added, and charges added where required) using the UCSF Chimera software (version 1.16) structure build module. Ligand binding site prediction was conducted by PrankWeb (http://prankweb.cz). The X-ray crystal structure of the iNOS with detailed resolution was obtained from Protein Data Bank (PDB) with PDB ID 1R35. The protein was docked with compounds using AutoDock Vina and UCSF Chimera, and the binding energies were calculated. The docking complexes were visualized using the ProteinPlus web server.

### 2.9. Statistical analysis

Statistical significances were determined by the one-way analysis of variance (ANOVA) and the Student's *t*-test. Data were expressed as mean ± SD of replicated experiments. The values of *P* < 0.05 were statistically significant.

## 3. Results and discussion

### 3.1. Characterization and identification of chemical constituents in LAE

In this study, UHPLC-HRESI-Q-orbitrap method was adopted to identify the chemical profiles in LAE, and the total ion chromatogram (TIC) under positive mode is shown in Fig. S1.† The compounds with available standards were identified by comparing the retention time and high-resolution accurate mass. Moreover, the MS fragmentation behaviors of the reference compounds have been previously reported in the literature, which was helpful for structural elucidation of the relative derivatives with the same skeleton.^11^ For compounds lacking available standards, the structures were tentatively identified by comparing with an in-house compounds database, according to the accurate mass, chromatographic behavior, MS/MS data, and fragmentation patterns. The mass errors for all the precursor ions of the identified compounds were set within ±5 ppm. Ultimately, a total of 80 compounds were unambiguously or tentatively identified, with sesquiterpenes and alkaloids comprising 60% of the total identified compounds. In the positive mode, the quasi-molecular ion peaks of alkaloids and sesquiterpenoids always appeared as [M + H]^+^ ions, and a series of fragmentation peaks such as [M + H − H_2_O]^+^, [M + H − CO]^+^, and [M + H − NH_3_]^+^ were observed in MS/MS spectra. A detailed information of these identified compounds is listed in Table 1, and the MS and MS/MS spectra of the identified compounds are provided in ESI.†

**Table 1:** Identification of compounds in LAE using UHPLC-HRESI-Q-orbitrap-MS in positive mode

Peak no.	*t*_R_ (min)	Molecular formula	Calculated mass (*m*/*z*)	Measured mass (*m*/*z*)	Reference ion	Error (ppm)	MS/MS fragments (*m*/*z*)	Identification	Class of compounds
1	7.94	C_17_H_17_NO_3_	283.1198	284.1271	[M + H]^+^	−3.58	145.0598, 178.0863, 223.0746, 255.1010	Norcinnamolaurine	Alkaloid
2	8.39	C_16_H_17_NO_3_	271.1199	272.1272	[M + H]^+^	−3.40	107.0495, 123.0445, 161.0603, 255.1022	Higenamine	Alkaloid
3	9.42	C_15_H_14_O_6_	290.0781	291.0853	[M + H]^+^	−3.40	123.0438, 139.0387, 147.0438, 165.0543	Catechin	Flavanol
4	9.57	C_11_H_9_NO_2_	187.0629	188.0702	[M + H]^+^	−2.33	118.0649, 146.0598, 170.0596	Indole-3-acrylic acid	Alkaloid
5	10.27	C_11_H_13_NO_2_	191.0943	192.1016	[M + H]^+^	−1.71	133.0646, 148.0754, 149.0833, 177.0781	Streptopyrrolidine	Alkaloid
6	10.43	C_6_H_11_NO	113.0839	114.0912	[M + H]^+^	−1.24	69.0698, 79.0541, 96.0806	Caprolactam	Amide
7	10.80	C_19_H_21_NO_4_	327.1462	328.1534	[M + H]^+^	−2.73	190.0859, 265.0849, 297.1109	Laurotetanine	Alkaloid
8	10.82	C_18_H_19_NO_3_	297.1356	298.1428	[M + H]^+^	−3.19	176.0701, 190.0853, 254.1168, 283.1195	Diolmycin A1	Alkaloid
9	11.53	C_18_H_19_NO_4_	313.1304	314.1377	[M + H]^+^	3.26	268.1323, 298.1054, 299.1142	Norbracteoline	Alkaloid
10	12.19	C_17_H_19_NO_3_	285.1357	286.1403	[M + H]^+^	−2.54	107.0489, 160.0754, 192.1016, 243.1009	Coclaurine	Alkaloid
11	12.83	C_10_H_11_NO_3_	193.0736	194.0810	[M + H]^+^	−1.39	107.0491, 119.0492, 135.0439, 151.0754	Northalifoline	Alkaloid
12	12.87	C_7_H_6_O_3_	138.0314	139.0387	[M + H]^+^	−1.88	93.0334, 111.0439, 139.0388	3-Hydroxy-2,5-toluquinone	Quinone
13	12.99	C_19_H_21_NO_4_	327.1460	328.1533	[M + H]^+^	−3.19	176.0701, 190.0855, 281.1040, 312.1221, 313.1300	*N*-Methylhernovine	Alkaloid
14	13.01	C_19_H_21_NO_3_	311.1512	312.1585	[M + H]^+^	−3.12	177.0906, 204.1015, 206.1171, 269.1163, 283.1318	Pronuciferine	Alkaloid
15	13.37	C_18_H_19_NO_4_	313.1305	314.1377	[M + H]^+^	−2.57	237.0904, 265.0851, 282.0877, 297.1112	Norisoboldine	Alkaloid
16	14.00	C_19_H_21_NO_4_	327.1460	328.1533	[M + H]^+^	3.20	237.0904, 265.0851, 297.1112	Boldine	Alkaloid
17	14.14	C_19_H_23_NO_4_	329.1617	330.1690	[M + H]^+^	−3.16	137.0595, 143.0489, 192.1015, 299.1267	Reticuline	Alkaloid
18	14.24	C_18_H_19_NO_4_	313.1305	314.1378	[M + H]^+^	−2.77	107.0489, 237.0903, 265.0851, 297.1112	Norboldine	Alkaloid
19	14.82	C_13_H_20_O_2_	208.1459	209.1532	[M + H]^+^	−1.89	95.0541, 133.1009, 135.0799, 153.0908, 191.1431	4,6,10,12-Tridecatetraene-2,8-diol	Aliphatic alcohol
20	15.92	C_19_H_21_NO_4_	327.1460	328.1533	[M + H]^+^	−3.23	192.1016, 233.0594, 265.0851, 282.0878	Isoboldine	Alkaloid
21	17.33	C_14_H_16_O_4_	248.1040	249.1113	[M + H]^+^	−3.44	105.0696, 159.0801, 187.0751, 204.0775	1-(5-Oxotetrahydrofuran-2-yl)ethyl-2-phenylacetate	Fruanone
22	17.40	C_20_H_23_NO_4_	341.1616	342.1689	[M + H]^+^	−3.23	248.0821, 279.1003, 296.1036, 311.1263	Isocorydine	Alkaloid
23	18.01	C_14_H_16_O_4_	248.1041	271.0933	[M + H]^+^	−2.92	91.0545, 105.0333	Epipyriculol	Phenolic derivative
24	18.58	C_20_H_23_NO_4_	341.1616	342.1689	[M + H]^+^	−3.23	237.0903, 265.0851, 280.1085, 296.1033	*N*-Methyllaurotetanine	Alkaloid
25	18.92	C_15_H_18_O_4_	262.1197	263.1270	[M + H]^+^	−3.15	105.0333, 107.0490	Linderolide S	Sesquiterpenoid
26	20.64	C_19_H_15_NO_5_	337.0940	338.1013	[M + H]^+^	−3.00	252.0648, 280.0593, 295.0827, 323.0778	9-Hydroxy-1,2,10-trimethoxy-7*H*-dibenzo[*de*,*g*]quinolin-7-one	Alkaloid
27	20.76	C_25_H_27_NO_5_	421.1883	422.1956	[M + H]^+^	−1.38	137.0595, 143.0489, 175.0750, 404.1843	Karakoramine	Alkaloid
28	20.81	C_18_H_21_NO_3_	299.1511	300.1584	[M + H]^+^	−3.35	107.0489, 151.0749, 174.0673, 189.0907, 283.1317	Norarmepavine	Alkaloid
29	20.88	C_15_H_18_O_4_	262.1196	285.1088	[M + Na]^+^	−3.35	105.0332, 107.0490, 165.0907, 241.1194	Linderolide D	Sesquiterpenoid
30	20.89	C_15_H_16_O_3_	244.1093	245.1166	[M + H]^+^	−2.57	107.0488, 189.1272, 199.1114, 217.1218	Neolinderalactone	Sesquiterpenoid
31	21.93	C_18_H_19_NO_4_	313.1303	314.1377	[M + H]^+^	−3.41	91.0539, 117.0333, 121.0646, 177.0543	*N*-Feruloyltyramine	Amide
32	22.13	C_15_H_18_O_5_	278.1145	279.1218	[M + H]^+^	−3.26	123.0829, 233.1167, 243.1009, 261.1124	Linderolide B	Sesquiterpenoid
33	22.90	C_15_H_18_O_3_	246.1247	247.1322	[M + H]^+^	−3.62	105.0336, 117.0697, 131.0855, 183.1165, 229.1219	Peniophoral	Aldehyde
34	24.67	C_15_H_18_O_3_	246.1249	247.1322	[M + H]^+^	−3.02	183.1165, 201.1271, 229.1217	8-Hydroxylindestenolide	Sesquiterpenoid
35	25.09	C_15_H_16_O_4_	260.1040	261.1113	[M + H]^+^	−3.51	95.0489, 215.1061, 233.1169, 243.1007	Parvigemone	Sesquiterpenoid
36	25.79	C_14_H_16_O_3_	232.1093	233.1166	[M + H]^+^	−2.77	105.0334, 145.1010, 173.0958, 215.1062	Citreoviripyrone B	Pyrone
37	26.11	C_15_H_18_O_4_	262.1197	285.1089	[M + Na]^+^	2.97	105.0333, 133.0644, 227.1066, 241.1192, 267.0984	Linderolide I	Sesquiterpenoid
38	26.40	C_15_H_20_O_4_	264.1353	287.1245	[M + Na]^+^	−3.32	105.0334, 133.0644, 149.0959, 269.1143	1-Methylabscisic-6-acid	Sesquiterpenoid
39	26.42	C_16_H_22_O_4_	278.1509	301.1401	[M + Na]^+^	−3.39	137.0571, 269.1142, 283.1295	2-Methyl butyl propyl phthalate	Phthalate esters
40	26.62	C_13_H_20_O	192.1511	193.1584	[M + H]^+^	−1.67	95.0853, 119.0853, 133.1010, 175.1478	β-Ionone	Terpenoid
41	26.73	C_15_H_18_O_2_	230.1300	231.1372	[M + H]^+^	−3.12	107.0853, 119.0853, 185.1322, 213.1269	Dehydrocostus lactone	Sesquiterpenoid
42	27.19	C_15_H_16_O_2_	228.1144	229.1217	[M + H]^+^	−2.57	119.0853, 131.0853, 159.0802, 201.1270	Lactarioline A	Sesquiterpenoid
43	27.64	C_15_H_24_	204.1873	205.1946	[M + H]^+^	−2.36	107.0853, 121.1166, 135.1167, 149.1323	α-Farnesene	Sesquiterpenoid
44	28.00	C_15_H_18_O_2_	230.1301	231.1373	[M + H]^+^	−2.73	93.0697, 133.0646, 203.1430, 213.1270	Lindestrenolide	Sesquiterpenoid
45	28.05	C_15_H_22_	202.1718	203.1791	[M + H]^+^	−1.88	105.0697, 119.0853, 133.1009, 147.1166	Calamenene	Sesquiterpenoid
46	28.16	C_13_H_16_O	188.1197	189.1270	[M + H]^+^	−2.09	105.0697, 131.0854, 143.0853, 174.1035	1-Phenylhept-3-en-4-one	Diarylheptanoid
47	28.29	C_14_H_14_O_2_	214.0989	215.1062	[M + H]^+^	−2.13	169.1010, 173.0968, 187.1114, 197.0958	2,4,6,8,10,12-Tetradeca-1,14-hexenedial	Aldehyde
48	28.30	C_15_H_16_O_4_	260.1040	261.1113	[M + H]^+^	−3.20	95.0490, 173.0958, 215.1063, 243.1009	Linderane	Sesquiterpenoid
49	28.45	C_13_H_22_O_2_	210.1614	228.1952	[M + NH_4_]^+^	−2.78	95.0854, 109.1010, 175.1478, 193.1584	10-Methyldodec-3-en-4-olide	Furanone
50	28.70	C_15_H_16_O_3_	244.1092	245.1165	[M + H]^+^	3.30	157.0654, 171.1173, 199.1124, 227.1072	Isolinderalactone	Sesquiterpenoid
51	28.74	C_15_H_18_O_2_	230.1301	231.1374	[M + H]^+^	−2.53	95.0490, 107.0853, 119.0854, 147.0801	Lindenenol	Sesquiterpenoid
52	28.74	C_16_H_20_O_2_	244.1456	245.1529	[M + H]^+^	−3.05	105.0697, 199.1113, 213.1269	Linderoxide	Sesquiterpenoid
53	28.76	C_14_H_20_O_2_	220.1456	243.1348	[M + Na]^+^	3.32	93.0697, 105.0697, 109.1010, 161.0595	Benquoine	Lactone
54	28.81	C_15_H_22_O	218.1666	219.1738	[M + H]^+^	−2.35	91.0541, 121.0642, 163.1113, 203.1427	Anaephene A	Alkylphenol
55	28.96	C_15_H_16_O_2_	228.1145	229.1217	[M + H]^+^	−2.51	105.0699, 131.0854, 201.0909, 211.1115	Dehydrolindestrenolide	Sesquiterpenoid
56	29.20	C_17_H_20_O_4_	288.1352	289.1425	[M + H]^+^	−3.19	105.0697, 183.1165, 211.1113, 229.1218	Lindenanolide A	Sesquiterpenoid
57	29.58	C_15_H_22_O_2_	234.1615	235.1688	[M + H]^+^	2.11	123.0441, 179.1063, 217.1579	Nigriterpene E	Sesquiterpenoid
58	30.52	C_15_H_18_O_2_	230.1299	253.1190	[M + Na]^+^	−3.45	147.0803, 159.1166, 175.0751, 185.1322	Shizukanolide A	Sesquiterpenoid
59	30.57	C_15_H_22_	202.1718	203.1791	[M + H]^+^	−1.88	105.0597, 119.0853, 133.1009, 147.1166	Rulepidadiene B	Sesquiterpenoid
60	31.03	C_17_H_16_O_5_	300.0997	301.1066	[M + H]^+^	−1.98	155.0488, 241.0856, 269.0804	Methyllinderone	Cyclopentenedione
61	31.27	C_15_H_18_O_2_	230.1301	231.1374	[M + H]^+^	−2.71	91.0841, 129.0694, 131.0854, 157.1010	Lorneic acid J	Acid
62	31.30	C_15_H_22_O	218.1666	219.1739	[M + H]^+^	−2.15	107.0855, 137.0956, 163.1113, 201.1426	α-Cyperone	Sesquiterpenoid
63	32.09	C_17_H_20_O_3_	272.1404	273.1478	[M + H]^+^	−3.07	105.0697, 157.1010, 185.0957, 213.1270	Lindenenyl acetate	Sesquiterpenoid
64	32.09	C_16_H_20_O_2_	244.1456	245.1529	[M + H]^+^	−2.86	105.0697, 157.1010, 185.1321, 213.1270	Isolinderoxide	Sesquiterpenoid
65	32.92	C_18_H_32_O_4_	312.2301	313.2369	[M + H]^+^	−2.50	183.0289, 245.0806, 269.0804	(2*E*,3*R*,4*S*)-2-Dodecylinene-3-hydroxy-4-ethoxy-4-methylbutanolide	Butanolide
66	32.97	C_15_H_18_O	214.1352	215.1425	[M + H]^+^	−2.45	95.0489, 109.0646, 159.0802, 173.1324	(4a*S*,8a*S*)-4,4a,5,6,8a,9-Hexahydro-3,8a-dimethyl-5-methylenenaphtho[2,3-*b*]furan	Furan
67	32.99	C_22_H_30_O_5_	374.2093	375.2162	[M + H]^+^	2.57	209.0809, 301.1795, 343.1895	1-Acetyl-4-methoxyl-denudaquinol	Phenolic derivative
68	33.03	C_16_H_14_O_5_	286.0841	287.0911	[M + H]^+^	−2.79	183.0289, 245.0806, 269.0804	Linderone	Cyclopentenedione
69	33.29	C_34_H_42_O_6_	546.2981	547.3034	[M + H]^+^	−1.57	201.1646, 329.1355, 370.8308, 424.9020	Linderin B	Sesquiterpenoid
70	33.63	C_16_H_33_NO	255.2554	256.2627	[M + H]^+^	−3.18	88.0755, 102.0911, 115.1057, 130.1225	Hexadecanamide	Amide
71	34.49	C_21_H_38_O_4_	354.2770	355.2838	[M + H]^+^	−1.27	109.0284, 127.0389, 268.9768	(2*E*,3*R*,4*S*)-2-Tetradecylinene-3-hydroxy-4-ethoxy-4-methylbutanolide	Butanolide
72	34.56	C_10_H_10_O_3_	178.0627	179.0700	[M + H]^+^	1.65	118.0412, 133.0645, 161.0595	Coniferyl aldehyde	Aldehyde
73	34.56	C_10_H_8_O_2_	160.0521	161.0594	[M + H]^+^	−1.80	103.0540, 105.0697, 118.0411, 133.0646	Ralfuranone A	Furanone
74	34.83	C_32_H_38_O_5_	502.2719	503.2793	[M + H]^+^	−2.48	273.1482, 379.1536, 393.1692	Lindechunisin A	Sesquiterpenoid
75	35.13	C_22_H_43_NO	337.3335	338.3407	[M + H]^+^	−3.00	163.1483, 177.1627, 303.3031, 321.3144	Erucamide	Amide
76	36.03	C_5_H_10_O	86.0730	87.0803	[M + H]^+^	−1.65	69.0699	Prenol	Terpenoid
77	36.26	C_10_H_16_O	152.1198	153.1271	[M + H]^+^	−1.88	97.0646, 107.0653, 109.1010, 135.1156	Isothujone	Terpenoid
78	36.49	C_16_H_32_O	240.2446	241.2519	[M + H]^+^	−2.91	69.0699, 83.0854, 97.1010, 111.1168	Hexadecan-2-one	Ketone
79	36.86	C_18_H_26_O_2_	274.1925	275.1998	[M + H]^+^	−2.76	91.0540, 117.0697 131.0853, 157.1009	Solwaric acid A	Aromatic acid
80	36.87	C_5_H_10_O	86.0731	87.0803	[M + H]^+^	−1.39	69.0699	Isoprenol	Terpenoid

**Fig. 1:**
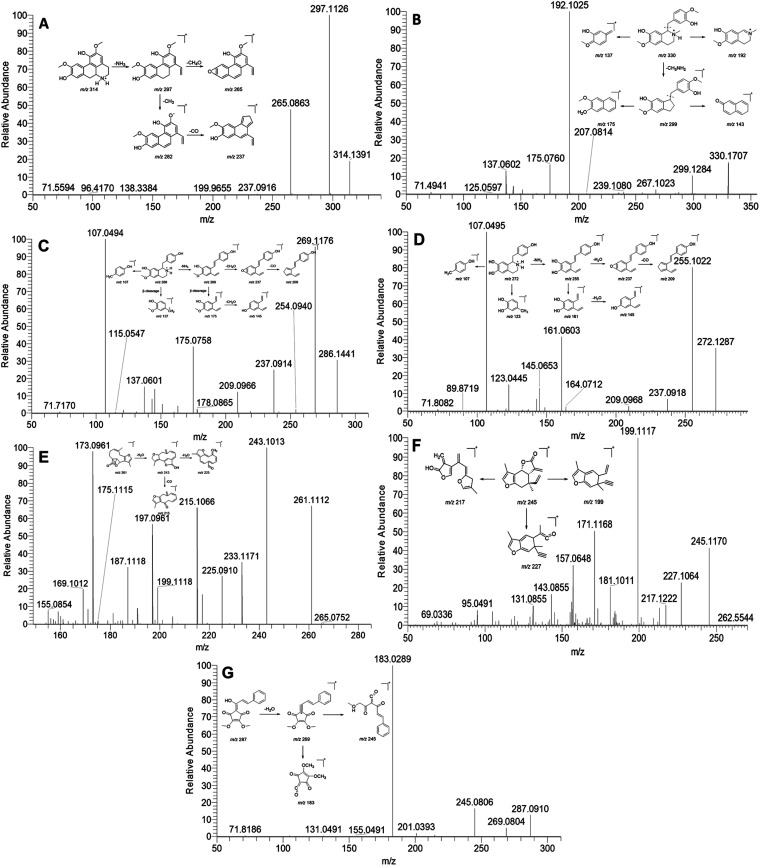
MS/MS spectra and the proposed fragmentation pathway of norisoboldine (A), reticuline (B), coclaurine (C), higenamine (D), linderane (E), isolinderalactone (F), and linderone (G).

Previous phytochemical investigations have demonstrated that isoquinoline alkaloids are one of the major components in *L. aggregata*. In this study, a total of 21 alkaloids were identified from LAE under the positive mode. Notably, the major ion peak 15 displayed a protonated molecular ion at *m*/*z* 314. Subsequent examination of the MS/MS spectra revealed two major product ion peaks at *m*/*z* 297 and 265 (Fig. 1A). The fragment peak at *m*/*z* 297 possibly resulted from the loss of an amino group, while the other fragment peak at *m*/*z* 265 arose from the loss of a methanol molecule from the ion *m*/*z* 297. Moreover, the product ion at *m*/*z* 297 was further fragmented to produce ions at *m*/*z* 282 and 237 due to consecutive losses of CH_3_ and CO, respectively. Based on these spectral characteristics, peak 15 was identified as norisoboldine and further confirmed by comparison with its authentic standard.^12^ Peaks 16, 18, and 21, exhibiting similar fragment patterns, were identified as boldine, norboldine, and isoboldine, respectively. Peak 17 showed a precursor ion at *m*/*z* 330, generating fragment ions at *m*/*z* 299 due to the loss of CH_3_NH_2_, alongside *m*/*z* 192 and 137, corresponding to the isoquinoline and the benzylic cleavage fragment, respectively. Additionally, the product ion peak at *m*/*z* 299 further produced ions at *m*/*z* 267 and 175, attributed to the loss of methanol and benzene moiety, respectively. Hence, peak 17 was confidently identified as reticuline and confirmed with the standard compound (Fig. 1B).^13^ Peak 10 displayed a protonated ion at *m*/*z* 286, generating a highly abundant product ion at *m*/*z* 269 through the loss of an amino group. Consecutive losses of CH_4_OH and CO resulted in fragment ions at *m*/*z* 237 and 209, respectively. Furthermore, a series of fragment ions at *m*/*z* 107, 137, 145, and 175, corresponding to β-cleavage of the skeleton, were detected. These characteristic fragment ions supported the identification of peak 10 as coclaurine, further confirmed by comparison with the standard compound (Fig. 1C).^13^ Similarly, peak 2 showed a comparable MS fragmentation pattern to peak 10. With a molecular weight that was 14 Da lower than that of peak 10, peak 2 was characterized as higenamine according to the proposed fragmentation pathway (Fig. 1D).^14^

Sesquiterpenoid is another major constituent in *L. aggregata*. A total of 27 sesquiterpenoids were either unambiguously or tentatively identified in LAE. The MS/MS spectra behaviors of sesquiterpenoids in *L. aggregata* are notably complex due to the presence of multiple sesquiterpenoid skeletons. Taking peak 48 as an example, it yielded a [M + H]^+^ ion at *m*/*z* 261. The MS/MS spectra showed main fragment ion peaks at *m*/*z* 243, 225, 215, and 197, which are attributed to the consecutive losses of H_2_O and CO. Consequently, peak 48 was unambiguously identified as linderane, and further confirmed using the standard compound (Fig. 1E). Peak 50 showed similar MS fragmentation characteristics to peak 48, and it was characterized as isolinderalactone according to the proposed fragmentation pathway (Fig. 1F). Based on these similar fragmentation patterns, peak 51 was identified as lindenenol.^15^

Peak 68 exhibited a precursor ion at *m*/*z* 287. The characteristic fragment ions observed at *m*/*z* 269, 245, and 183 were attributed to the loss of H_2_O and the cleavage of the skeleton, leading to the identification of this peak as linderone (Fig. 1G). Peak 60 displayed a similar MS fragmentation pattern with peak 68, with a molecular weight that is 14 Da higher than that of peak 68. Thus, peak 60 was characterized as methyllinderone.^16^ Both the above cylcyclopentenediones were confirmed with standard compounds. Additionally, some interesting compounds (including peaks 67, 69, 71, and 74) were first identified from *L. aggregata* and confirmed with standard compounds. Based on the above results, the 16 compounds (including higenamine (2), coclaurine (10), norisoboldine (15), boldine (16), reticuline (17), linderane (48), isolinderalactone (50), lindeneol (51), methyllinderone (60), lindenenyl acetate (63), (2*E*,3*R*,4*S*)-2-dodecylinene-3-hydroxy-4-ethoxy-4-methylbutanolide (65), 1-acetyl-4-methoxyl-denudaquinol (67), linderone (68), linderin B (69), (2*E*,3*R*,4*S*)-2-tetradecylinene-3-hydroxy-4-ethoxy-4-methylbutanolide (71), and lindechunisin A (74)) were selected for further quantification analysis.

### 3.2. Quantitative analysis of the selected compounds

**Fig. 2:**
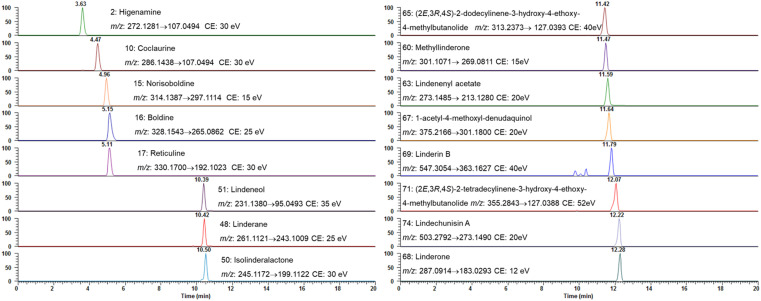
Extracted ion chromatograms of the quantified analytes in PRM mode.

Based on the qualitative results, the aforementioned 16 compounds were selected for quantitative analysis due to their predominance in the chemical profile of *L. aggregata* and their established or potential bioactivities. These compounds, including some key alkaloids and sesquiterpenoids, are critical for assessing the plant's quality. Herein, an UHPLC-MS/MS quantification method using PRM mode was established. The MS/MS detection parameters, such as ion pairs and collision energy, were optimized by directly injecting each analyte to achieve the most sensitive and stable reaction monitoring transitions. Fig. 2 displayed the PRM extracted ion chromatogram for each compound.

The stock solution was diluted with LC-MS-grade solvent for different working concentrations to establish the calibration curves. The calibration curves were constructed using a series of different concentrations by least-square linear regression analysis. The linearity of each compound's calibration curve was assessed through repeated experiments, employing the regression coefficient (*r*^2^) within the tested concentration ranges (Table 2). Results demonstrated that good linearity was achievable within the tested concentration ranges. The LODs and LOQs fell within the range of 0.033–0.33 ng mL^−1^ and 0.11–1.1 ng mL^−1^, respectively. The RSD for precision and reproducibility were in the range of 1.0–4.2%, and 0.9–5.6%, respectively. The recovery rate of the analytes ranged from 85.4% to 114.7%. All the above results indicated that the analysis method was valid and reliable. Based on the established analysis method, the average content of each compound was determined as follows: higenamine was 0.39 ± 0.02 mg g^−1^, coclaurine was 0.95 ± 0.15 mg g^−1^, norisoboldine was 50.34 ± 4.27 mg g^−1^, boldine was 6.53 ± 0.24 mg g^−1^, reticuline was 3.30 ± 0.16 mg g^−1^, linderane was 23.47 ± 0.18 mg g^−1^, isolinderalactone was 10.45 ± 0.88 mg g^−1^, lindeneol was 16.70 ± 1.17 mg g^−1^, methyllinderone was 0.10 ± 0.01 mg g^−1^, lindenenyl acetate was 11.57 ± 0.08 mg g^−1^, (2*E*,3*R*,4*S*)-2-dodecylinene-3-hydroxy-4-ethoxy-4-methylbutanolide was 0.09 ± 0.01 mg g^−1^, 1-acetyl-4-methoxyl-denudaquinol was 0.28 ± 0.02 mg g^−1^, linderone was 0.14 ± 0.01 mg g^−1^, linderin B was 0.55 ± 0.03 mg g^−1^, (2*E*,3*R*,4*S*)-2-tetradecylinene-3-hydroxy-4-ethoxy-4-methylbutanolide was 0.17 ± 0.01 mg g^−1^, and lindechunisin A was 0.15 ± 0.01 mg g^−1^.

**Table 2:** Validation results of the developed quantification method of 16 compounds (*n* = 6)

No.	Compounds	Linearity			LOD (ng mL^−1^)	LOQ (ng mL^−1^)	Precision RSD (%)	Reproducibility RSD (%)	Recovery (%)
		Linear range (ng mL^−1^)	*r* ^2^	Regression equation					
2	Higenamine	2.65–133.00	0.9997	*Y* = −115 619 + 3 219 43*x*	0.086	0.273	1.5	2.8	94.1–114.7
10	Coclaurine	2.50–125.00	0.9998	*Y* = 466 122 + 4 807 91*x*	0.033	0.109	1.3	3.5	91.0–103.6
15	Norisoboldine	2.45–245.00	0.9999	*Y* = 371 228 + 6 331 45*x*	0.079	0.235	1.9	3.0	85.4–98.5
16	Boldine	2.50–125.00	0.9997	*Y* = 753 908 + 8 390 67*x*	0.040	0.133	1.1	3.7	94.6–107.3
17	Reticuline	2.38–119.00	0.9995	*Y* = 84 052 + 11 235 80*x*	0.032	0.110	1.0	3.7	95.2–114.7
48	Linderane	10.56–1056.00	0.9995	*Y* = 1745.7 + 2941.5*x*	0.308	1.062	1.6	5.6	96.7–108.5
50	Isolinderalactone	9.82–982.00	0.9994	*Y* = −824 931 + 1 000 49*x*	0.291	0.975	2.0	3.7	90.1–109.8
51	Lindeneol	10.80–540.00	0.9994	*Y* = −59 915.2 + 8850.63*x*	0.317	1.078	1.9	4.1	85.9–97.9
60	Methyllinderone	2.31–116.00	0.9998	*Y* = −2148.54 + 2 298 64*x*	0.077	0.228	4.2	1.7	95.3–98.9
63	Lindenenyl acetate	10.61–1061.00	0.9999	*Y* = −4263.81 + 881.66*x*	0.152	0.527	2.6	3.5	91.5–113.9
65	(2*E*,3*R*,4*S*)-2-Dodecylinene-3-hydroxy-4-ethoxy-4-methylbutanolide	10.61–1061.00	0.9999	*Y* = −21 702.4 + 644.61*x*	0.346	1.125	4.1	2.2	96.0–101.5
67	1-Acetyl-4-methoxyl-denudaquinol	2.50–125.00	0.9998	*Y* = −79 756 + 3264.71*x*	0.084	0.253	1.8	4.2	97.3–103.7
68	Linderone	2.34–117.00	0.9994	*Y* = −100 140 + 1 480 63*x*	0.078	0.228	2.4	0.9	97.5–103.0
69	Linderin B	2.48–245.00	0.9999	*Y* = 27 886.34 + 1974.69*x*	0.082	0.251	1.2	2.1	93.2–98.5
71	(2*E*,3*R*,4*S*)-2-Tetradecylinene-3-hydroxy-4-ethoxy-4-methylbutanolide	9.82–982.00	0.9994	*Y* = −32 503.2 + 617.441*x*	0.334	0.980	1.2	1.1	91.1–100.1
74	Lindechunisin A	2.65–133.00	0.9998	*Y* = 17 787.3 + 1585.95*x*	0.088	0.272	2.2	3.0	96.3–102.5

### 3.3. Anti-inflammatory effects of LAE and selected compounds

#### 3.3.1. Effect of LAE and compounds on the viability of RAW 264.7 cells

RAW 264.7 cells were exposed to various concentrations of LAE and compounds for 24 h, and the cell viability was detected using the MTT method. The assay results showed that LAE did not display remarkable cytotoxicity on RAW 264.7 cells at the concentration of 100 μg mL^−1^. Therefore, subsequent experiments were conducted at LAE concentrations up to 100 μg mL^−1^. Meanwhile, compounds 2, 10, 15, 16, 17, 48, 50, 51, 60, 63, 65, 67, 68, 69, 71, and 74 (which were obtained commercially or isolated from *L. aggregata*) did not change the cell viability at the concentration ranges of 0–100 μM. Thus, 6.25, 12.5, 25, 50, and 100 μM were adopted as the test concentrations of the selected 16 compounds, respectively.

#### 3.3.2. NO and iNOS protein production inhibitory effect assay

**Fig. 3:**
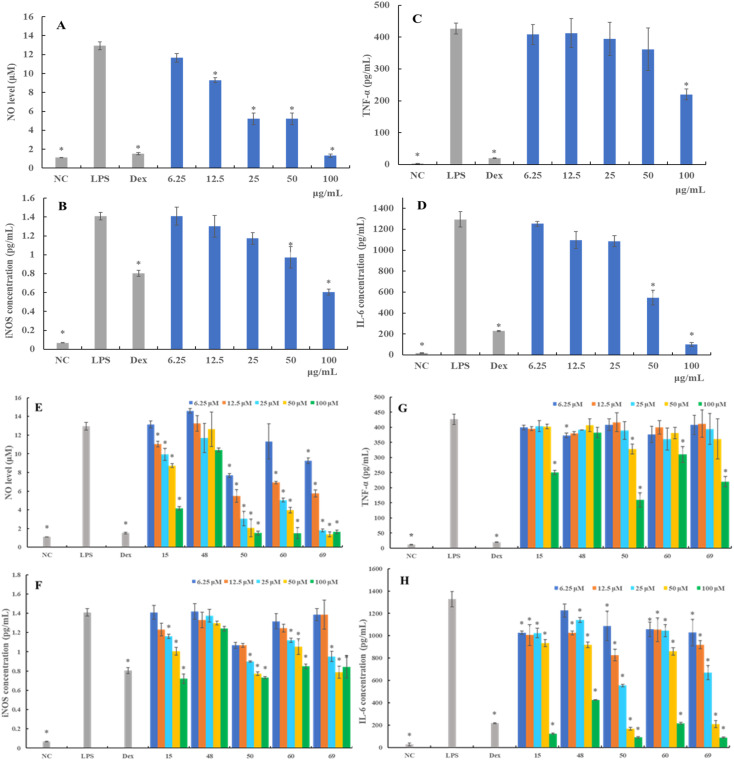
The effects of LAE on the production of NO (A), iNOS (B), TNF-α (C), and IL-6 (D) in LPS-induced RAW264.7 cells. The effects of norisoboldine (15), linderane (48), isolinderalactone (50), methyllinderone (60), and linderin B (69) on the production of NO (E), iNOS (F), TNF-α (G), and IL-6 (H) in LPS-induced RAW264.7 cells. RAW 264.7 cells were treated with various concentrations of LAE and selected compounds in the presence of LPS (1 μg mL^−1^) for 24 h. Protein expression of iNOS, TNF-α, and IL-6 in the culture medium was assayed by ELISA. NC: negative control group, LPS: LPS-treated group, Dex: dexamethasone-treated group. Data are presented as mean ± SD (*n* = 3). *p > 0.05, compared with the LPS-treated group.

As an initial preliminary screening, we tested the inhibitory capacity of LAE against NO and iNOS production at nontoxic concentrations in LPS-induced RAW 264.7 cells. As given in Fig. 3A and B, LAE inhibited NO and iNOS production in a dose-dependent manner. At the concentration of 100 μg mL^−1^, LAE reduced NO and iNOS production by 89.79% and 56.81%, respectively, compared with LPS-induced group. These results suggested that LAE inhibited the release of NO by suppressing iNOS protein expression in LPS-induced RAW264.7 cells. To elucidate the active constituents in LAE, 16 selected compounds were evaluated for inhibitory effect. Following preliminary screening, it was discerned that four compounds (15, 50, 60, and 69) demonstrated inhibitory effects on the generation of NO and iNOS. Fig. 3E illustrates that the treatment with LPS (1 μg mL^−1^) notably elevated the NO level in the culture supernatant of RAW 264.7 cells. Conversely, the treatment with over 12.5 μM of compounds 15, 50, 60, and 69 significantly suppressed NO production in a dose-dependent manner in RAW 264.7 cells stimulated with LPS. Additionally, compounds 15, 50, 60, and 69 at 100 μM exhibited remarkable reduction in NO formation by 67.86%, 88.26%, 88.38%, and 87.18%, respectively, compared to LPS-stimulated control cells.

iNOS mediates inflammatory reactions and catalyzes the synthesis of NO.^17^ Therefore, we further examined the alternation of iNOS protein production following different treatments. As shown in Fig. 3F, the expression of levels of iNOS in RAW 264.7 cells substantially increased upon LPS stimulation. Compounds 15, 50, 60, and 69 demonstrated a concentration-dependent attenuation of LPS-induced iNOS expression, mirroring its effects on NO production. More specifically, compounds 50, 60, and 69 showed stronger inhibitory activity against iNOS production than compound 15.

#### 3.3.3. Effect of LAE and compounds on cytokines production in RAW 264.7 cells

**Fig. 4:**
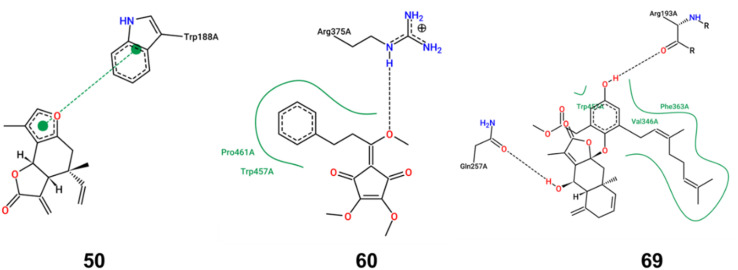
Docking model of compounds 50, 60, and 69 with iNOS. The interaction patterns are composed of hydrogen bonds, displayed as black dashed lines; π interactions, shown as green dashed lines with dots denoting the participating π systems; and hydrophobic contacts, which are represented by the residue labels and spline segments along the contacting hydrophobic ligand's part.

Next, we measured the effect of LAE and selected compounds on the production of TNF-α and IL-6 in LPS-treated RAW 264.7 cells *via* ELISA. As displayed in Fig. 3C and 4D, LPS stimulation for 24 h led to remarkable increase of TNF-α and IL-6 levels in the cell supernatants. The LPS-induced increases of TNF-α and IL-6 were dose-dependently reversed by LAE treatment. At the concentration of 100 μg mL^−1^, the release of TNF-α and IL-6 was reduced by up to 48.43% and 92.29%, respectively, compared to LPS-stimulated control cells.

Regarding compound evaluation, compounds 15, 50, 60, and 69 were found to significantly inhibit the expression of TNF-α at the concentration of 100 μM, and the TNF-α level decreased by 41.23%, 62.48%, 27.29%, and 48.43%, respectively (Fig. 3G). However, compound 48 showed a relatively weaker inhibitory effect compared to compounds 15, 50, 60 and 69, and did not exhibit a significant dose–response relationship. Further, the expression of IL-6 decreased dose-dependently following treatment with compound 50, which was as effective as compound 69, and even more effective than compounds 15, 48, and 60 (Fig. 3H). These results suggested that LAE and compounds 15, 48, 50, 60, and 69 suppressed the production of pro-inflammatory cytokines in the LPS-stimulated RAW 264.7 cells, which might be associated with the anti-inflammatory activity.

### 3.4. Molecular docking studies

iNOS can be activated by cytokines or LPS, leading to substantial NO secretion and subsequent inflammation. To elucidate the inhibitory mechanisms of the bioactive compounds, molecular docking studies were performed with iNOS. Compounds 50, 60, and 69, which demonstrated a significant inhibition effect on iNOS expression, were selected as the ligands. As shown in Fig. 4, compound 50 primarily exhibited π–π stacking interactions with iNOS, suggesting a strong affinity and potential specificity towards the enzyme's active site. In contrast, compounds 60 and 69 primarily engaged in hydrogen bond and hydrophobic interactions with iNOS. The binding energy score value was −7.7, −6.1, and −9.2, respectively. Owing to the lack of hydroxyl groups in compound 50, it is incapable of forming hydrogen bonds with iNOS. Consequently, the unsaturated lactone moiety formed a π–π interaction with the Trp188 residue of iNOS. Such π–π interaction, recognized as the most prevalent noncovalent interaction, manifests as favorable forces between the aromatic subunits of the biochemical molecules.^18^ The aromatic side chains of the amino acids, tryptophan and phenylalanine, are commonly modeled with indole and benzene, respectively. The π–π interaction, characterized by its broad and large surface area of contact, typically results in high binding energy. This implies that a protein–ligand complex can exhibit a high binding affinity even in the absence of hydrogen bonds. In the complex where iNOS interacted with compound 60, a single hydrogen bond was formed with Arg375, accompanied by hydrophobic interactions involving Pro461 and Trp457. Similarly, the iNOS complex with compound 69 established two hydrogen bonds with Arg193 and Gln257 and hydrophobic interactions with Trp450, Phe363 and Val346. While hydrogen bonds are relatively weaker than covalent or ionic bonds, their collective contribution can be substantial in terms of the overall binding energy between a ligand and its receptor. The oxygen atoms in methoxy group (compound 60) and hydroxy groups (compound 69) acted as hydrogen bond acceptors, significantly influencing the iNOS inhibitory activity. Moreover, the benzene rings and geranyl group in compounds 60 and 69 were likely to engage in robust interactions with the hydrophobic amino acid residue. These interactions were pivotal in enhancing the stability of the ligand–enzyme complex. Collectively, these findings provided a theoretical rationale for the effective binding of compounds 50, 60, and 69 to the inflammatory target iNOS, thereby inhibiting the expression of inflammatory markers.

*L. aggregata* is a widely used traditional Chinese medicine and new food resource with reported curative effects in various aspects, including anti-cancer, anti-arthritis, anti-bacterial, anti-oxidation, anti-diabetic nephropathy, hepatoprotective, and lipid-lowering effects. Owing to its significant medicinal value and wide-ranging pharmacological applications, *L. aggregata* has attracted increasing attention in China. This herb is found across various regions in China, with specimens from Zhejiang Province being particularly esteemed for their quality.^2^ However, a notable challenge in the cultivation and utilization of *L. aggregata* is the genetic diversity and variation in the medicinal component content across different geographical locations. This variability poses a significant challenge to the consistency and stability of *L. aggregata*'s quality, representing a crucial bottleneck in the standardization of its cultivation. This highlights the need for more focused research and development efforts to standardize and optimize the cultivation practices for *L. aggregata*, ensuring uniformity in the quality of this important medicinal plant. Although over 250 compounds have been isolated from *L. aggregata* to date, quantitative studies on its roots are relatively limited. Only nine compounds, including five sesquiterpenes and four alkaloids, have been quantified using LC-MS method.^7,8^ In this study, 80 compounds were identified, and 16 of them were quantified using LC-MS/MS method. Besides expanding the quantitative analysis of alkaloids and sesquiterpenoids in *L. aggregata*, butanolides and acyclopentendiones were quantified in *L. aggregata* for the first time, further advancing the quantitative study of *L. aggregata*. Given the comprehensive nature of the research on the anti-inflammatory effects of *L. aggregata*, we employed an approach that integrates both qualitative and quantitative methodologies. Our emphasis was on expeditiously identifying and quantifying anti-inflammatory compounds. The findings revealed that compounds 15, 50, 60, and 69 exhibited notable anti-inflammatory properties in LPS-induced RAW 264.7 cells, specifically evidenced by its ability to inhibit the production of NO and iNOS, alongside a marked suppression of cytokines including TNF-α, and IL-6. Extensive previous research has reported norisoboldine (15) as an anti-inflammatory property, demonstrating its capability to reduce systemic inflammation. Studies have demonstrated that norisoboldine possessed the capability to inhibit the production of pro-inflammatory factors and down-regulate the activation of MAPKs in LPS-induced RAW 264.7 cells.^19^ As a result of these established properties, norisoboldine has been designated as the chemical marker for quality evaluation in China Pharmacopoeia. Compound 50, isolinderalactone, is a representative elemane-type sesquiterpene lactone from *L. aggregata*. Molecular docking results suggested the unsaturated lactone moiety in compound 50 probably contributed significantly to its anti-inflammatory effect. This observation was echoed in the findings of Shen *et al.*, who also identified the unsaturated lactone moiety as critical for the compound's anti-inflammatory effects.^20^ Notably, they observed that the anti-inflammatory efficacy of isolinderalactone was almost completely diminished when the unsaturated double bond was reduced, further underscoring the importance of this structural feature.^20^ Compound 60 (methyllinderone) is a rare natural acyclopentendione. Its anti-inflammatory activity was intimately associated with the presence of the methoxy group based on the molecular docking results, potentially elucidating its better activity compared with linderone. Compound 69 (linderin B), comprising a sesquiterpenoid lactone and a methyl geranylhomogentisate moiety, was identified as a new compound in our previous study.^21^ Although characterized by a complex conjugate, the presence of a long-chain geranyl group probably enhanced its hydrophobic interactions with iNOS. Building upon the results, we conducted quantitative studies on these compounds, aiming to provide a basis for quality control enhancement. The anti-inflammatory compounds in *L. aggregata* are crucial to its medicinal properties, traditionally used for treating inflammation-related ailments like arthritis and gastrointestinal disorders. These phytochemicals, particularly sesquiterpenes and alkaloids, could inhibit pro-inflammatory mediators such as cytokines and enzymes, reducing inflammation. This makes L.*aggregata* effective in treating chronic inflammatory conditions and supports its therapeutic potential in modern medicine. Scientific studies have validated these effects, reinforcing the plant's relevance as a treatment for inflammation in both traditional and contemporary pharmacology. In summary, *L. aggregata*'s diverse bioactive compounds work synergistically, offering a broad therapeutic spectrum through multi-ingredient interactions. Future research will focus on standardizing extracts, exploring additional bioactivities, and elucidating the molecular mechanisms underlying its effects.

## 4. Conclusions

In conclusion, the chemical profiling and identification of 80 compounds in LAE, followed by quantification of 16 compounds, based on LC-MS/MS method were introduced herein. The quantification results might be used as chemical markers for the quality control of *L. aggregata*. This study further showcased the capability of LAE and some specific constituents in effectively suppressing NO, iNOS, TNF-α, and IL-6 in LPS-induced RAW 264.7 cells. Molecular docking investigations have provided insights into the mechanisms underlying the iNOS inhibitory activities of specific compounds. These results will hold significance for the enhancement of quality control and assurance processes for *L. aggregata* and related functional products, contributing to a better understanding of their potential health-promoting properties.

## Data availability

The authors confirm that the data supporting the results of this study are available within the article and its ESI.†

## Conflicts of interest

The authors declare no conflict of interest.

## Supplementary Material

RA-014-D4RA05643D-s001
