# Supplementary material for: Characterization and quantification of the phytochemical constituents and anti-inflammatory properties of Lindera aggregata
Source: RSC Adv. 2024 Nov 11;14(48):36101–14. doi: 10.1039/d4ra05643d (PMC11551842; doi:10.1039/d4ra05643d)

Supplementary Information (SI) for RSC Advances.
This journal is © The Royal Society of Chemistry 2024

Supplementary Material

Characterization and quantification of phytochemical constituents and anti-inflammatory properties of *Lindera aggregata*

Song-Song Wen[a,b], Yong-Jun Liu[a], Yu-Lin Liu[c], Yan Zhao[b], Xiao-Xi Xu[d], Chang-Chuan Guo[b], Chong Niu[b], Wei-Jian Wang[b], Yu-Wen Xu[b*], and Na Zhang[a*]

[a]Department of Pharmaceutics, Key Laboratory of Chemical Biology (Ministry of Education), School of Pharmaceutical Sciences, Cheeloo College of Medicine, Shandong University, Jinan, 250012, China

[b]NMPA Key Laboratory for Research and Evaluation of Generic Drugs, Shandong Research Center of Engineering and Technology for Consistency Evaluation of Generic Drugs, Shandong Institute for Food and Drug Control, Jinan, 250101, China

[c]Department of Marine Pharmacology, College of Food Science and Technology, Shanghai Ocean University, Shanghai, 201306, China

[d]Beijing Normal University, Beijing, 100875, China

*Corresponding author

Na Zhang, Department of Pharmaceutics, Key Laboratory of Chemical Biology (Ministry of Education), School of Pharmaceutical Sciences, Cheeloo College of Medicine, Shandong University, E-mail: zhangnancy9@sdu.edu.cn; Yu-Wen Xu, NMPA Key Laboratory for Research and Evaluation of Generic Drugs, Shandong Research Center of Engineering and Technology for Consistency Evaluation of Generic Drugs, Shandong Institute for Food and Drug Control, E-mail: 13553158409@163.com.

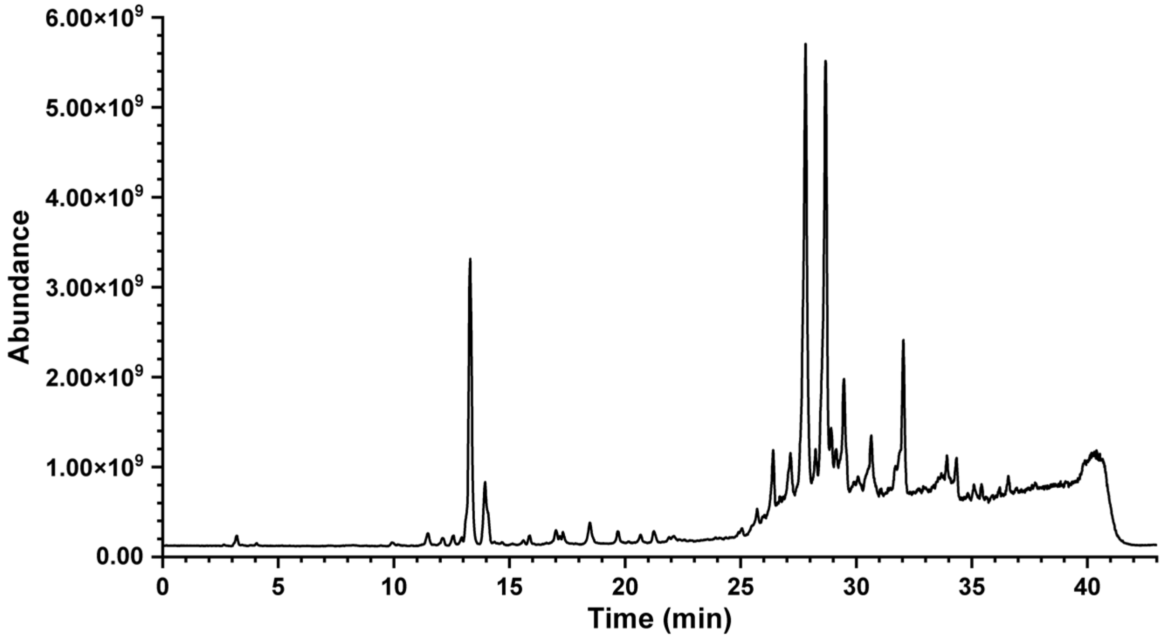

Figure S1. Total ion chromatogram (TIC) of LAE

MS and MS/MS spectra of identified compounds

7.937 min Norcinnamolaurine

MS

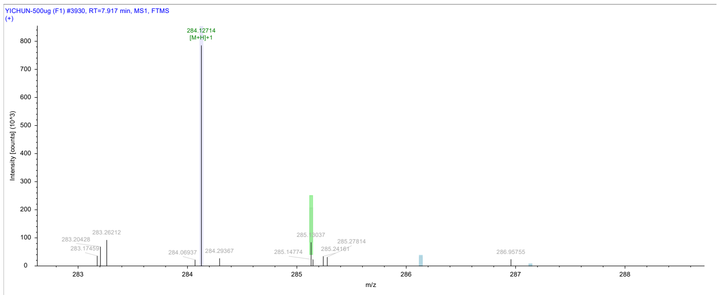

MS/MS

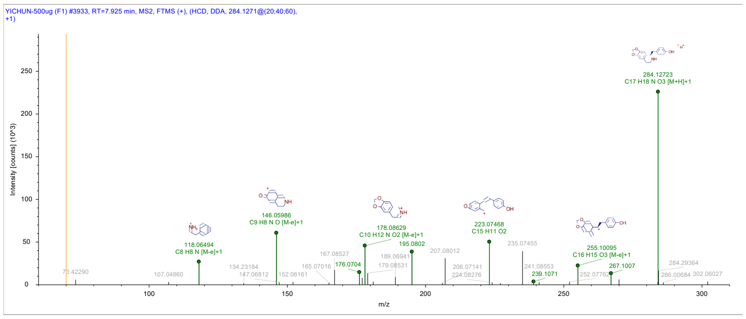

8.39 min Higenamine

MS

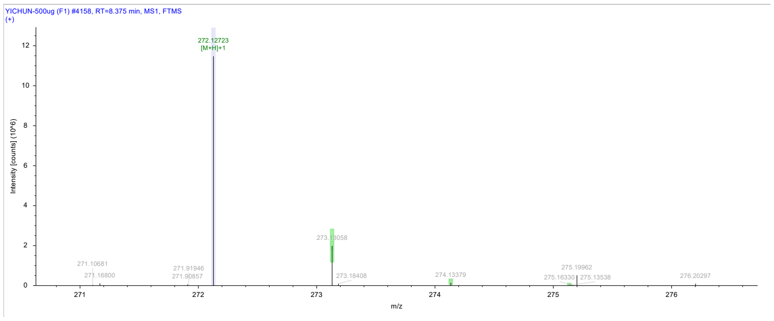

MS/MS

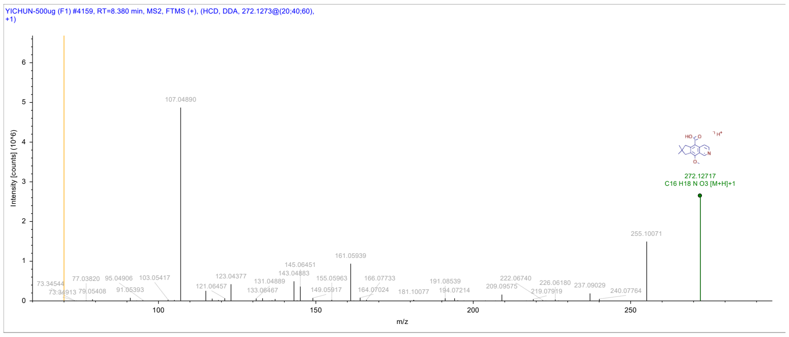

9.424 min Catechin

MS

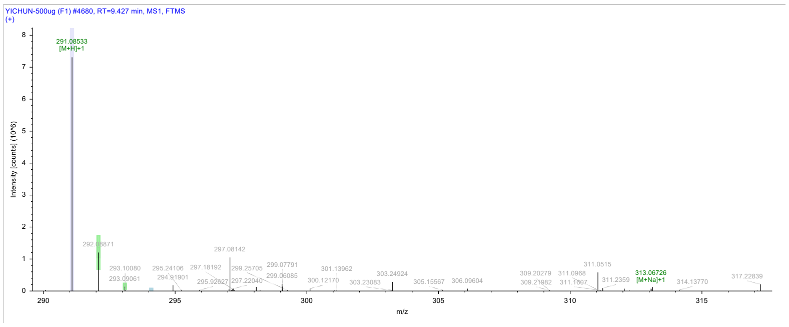

MS/MS

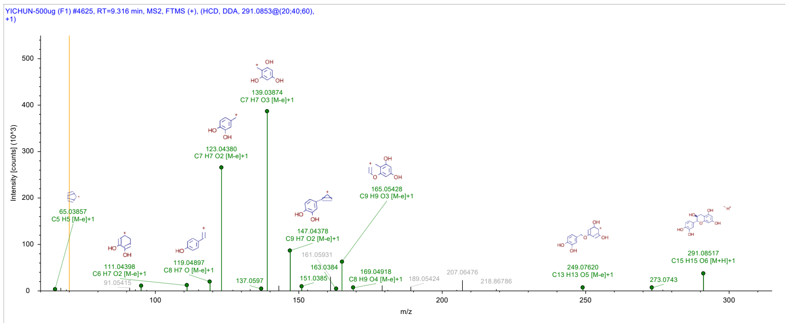

9.569 min Indole-3-acrylic acid

MS

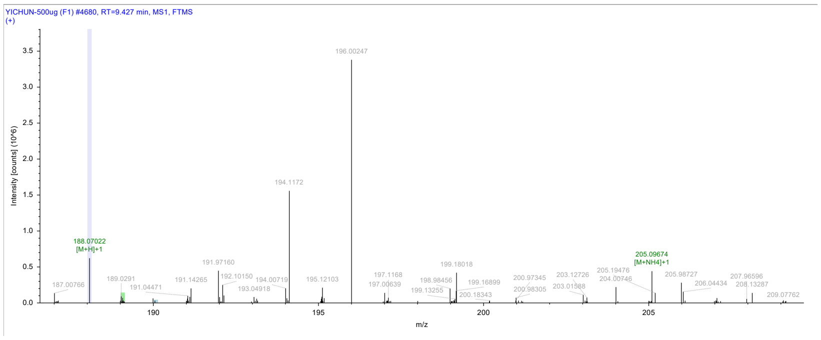

MS/MS

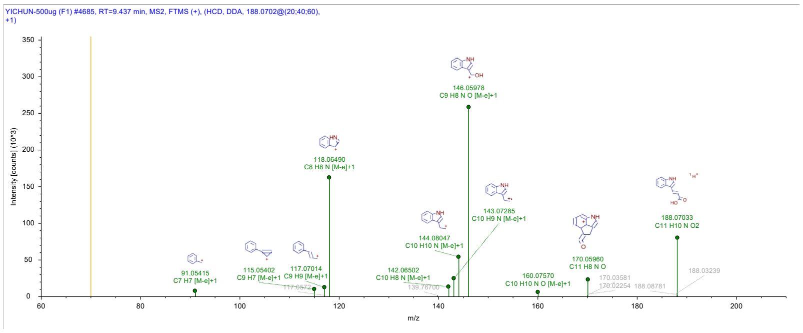

10.271 min Streptopyrrolidine

MS

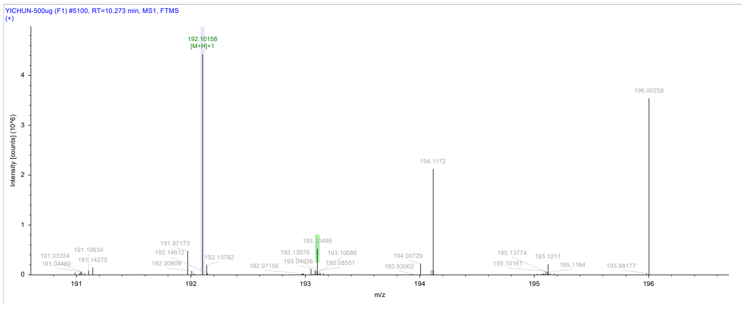

MS/MS

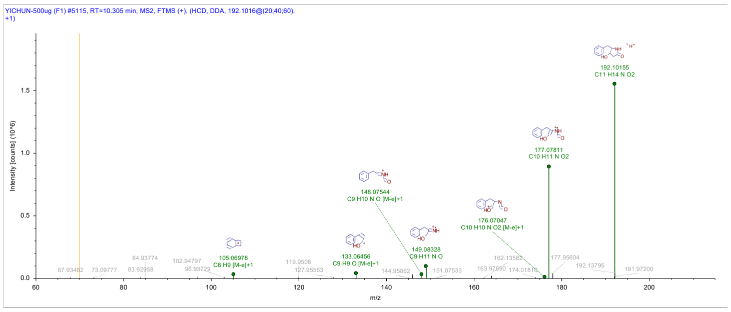

10.433 min Caprolactam

MS

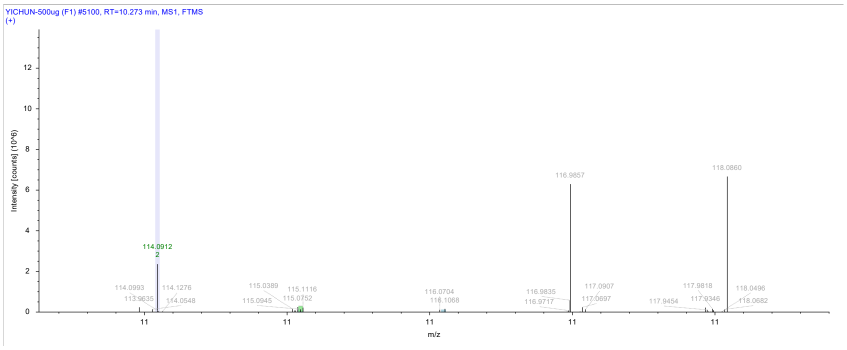

MS/MS

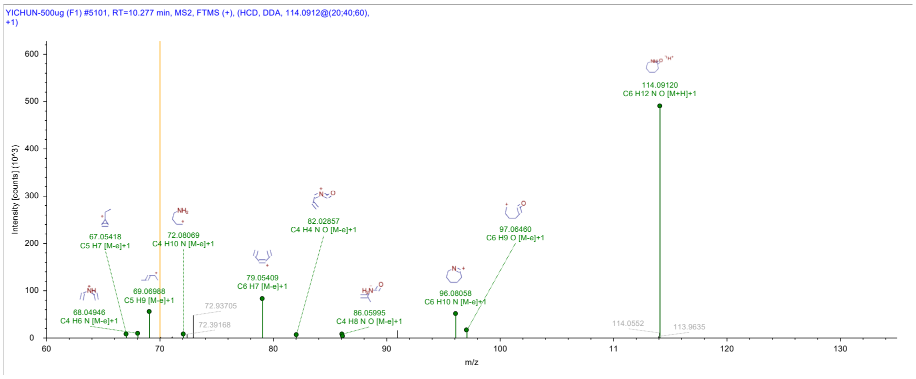

10.802 min Laurotetanine

MS

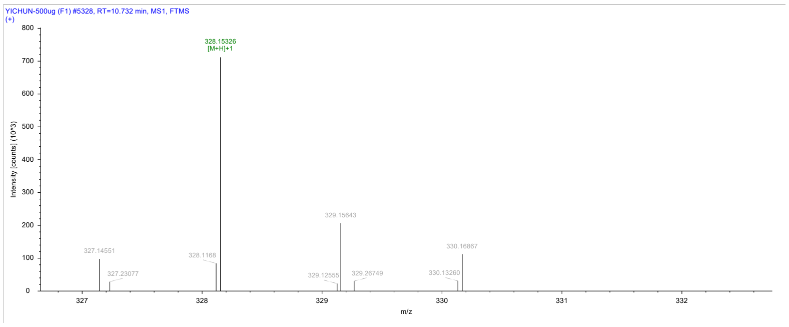

MS/MS

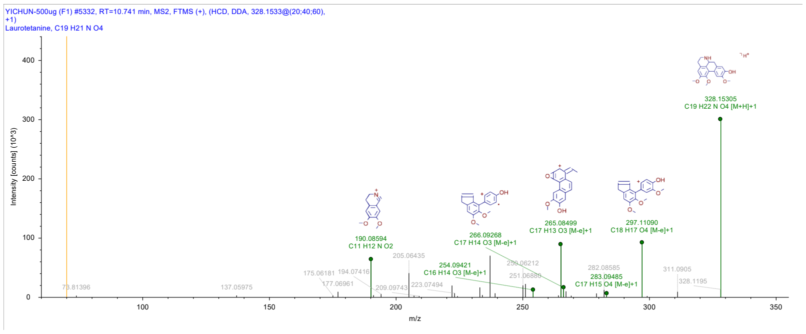

10.82 min Diolmycin A1

MS

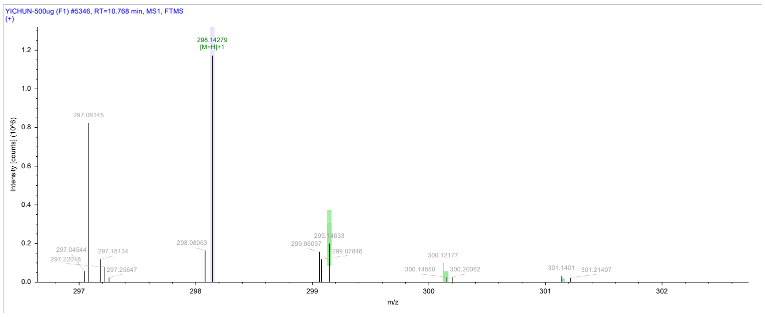

MS/MS

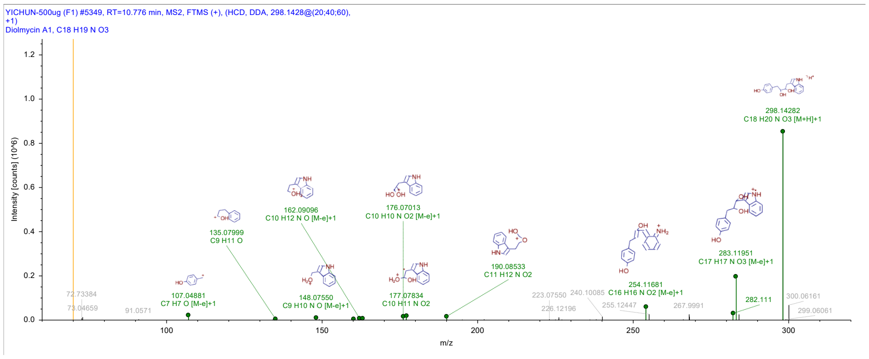

11.53 min Norbracteoline

MS

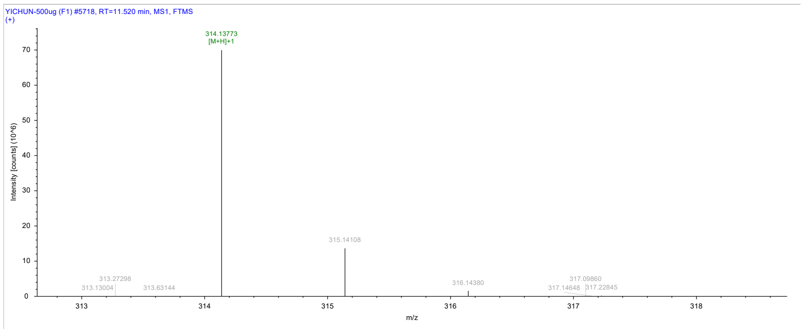

MS/MS

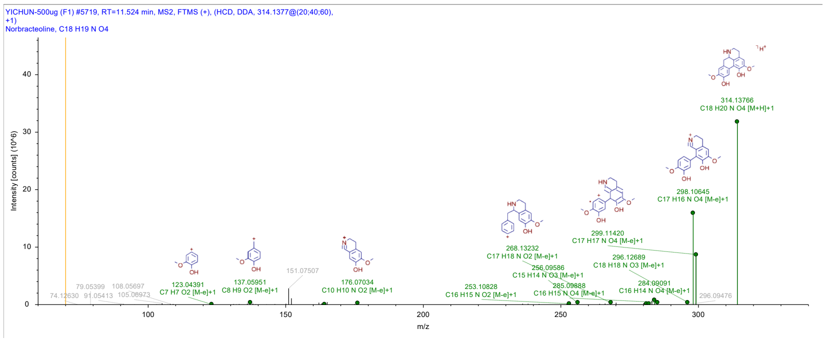

12.19 min Coclaurine

MS

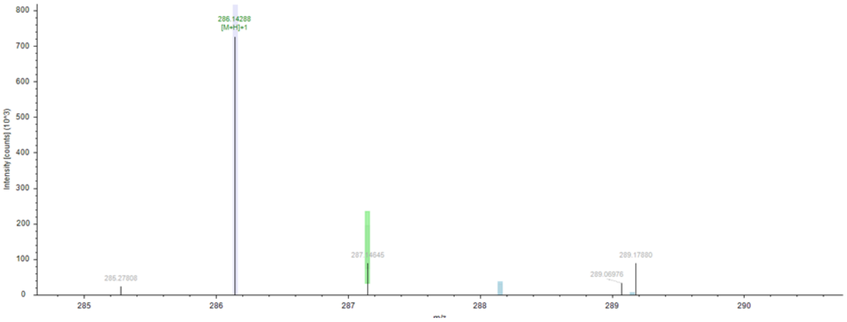

MS/MS

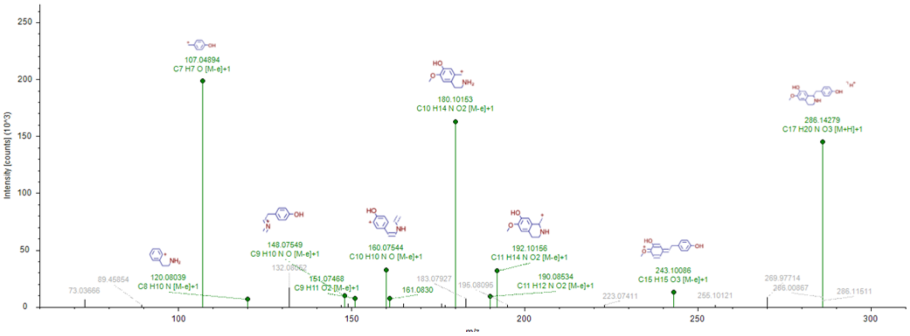

13.371 min Norisoboldine

MS

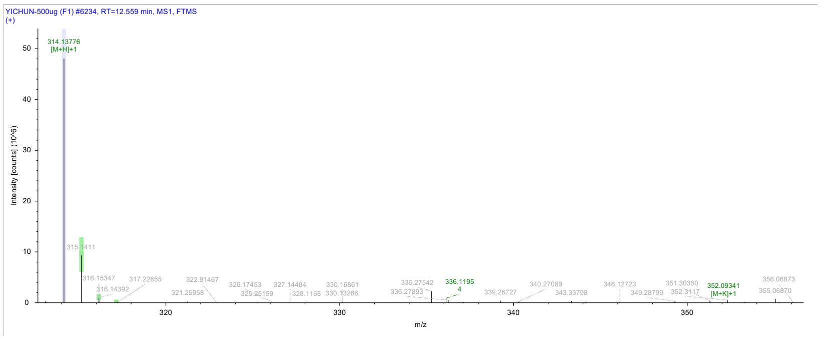

MS/MS

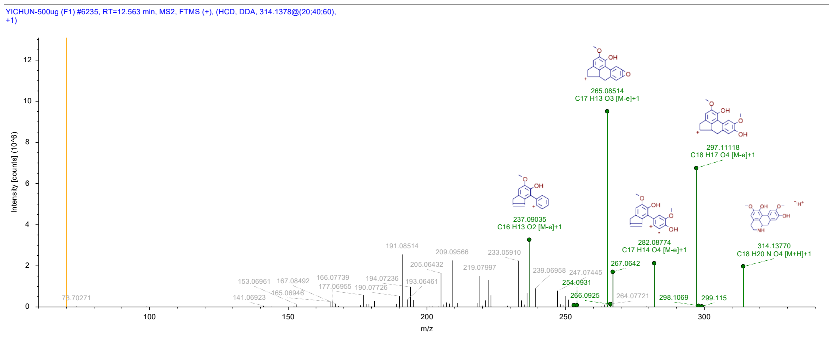

12.83 min Northalifoline

MS

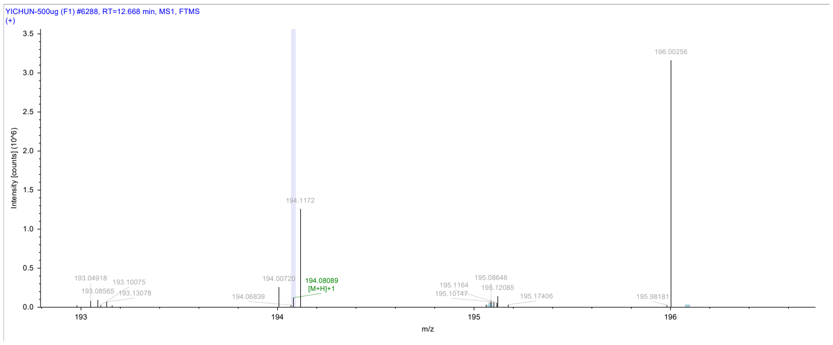

MS/MS

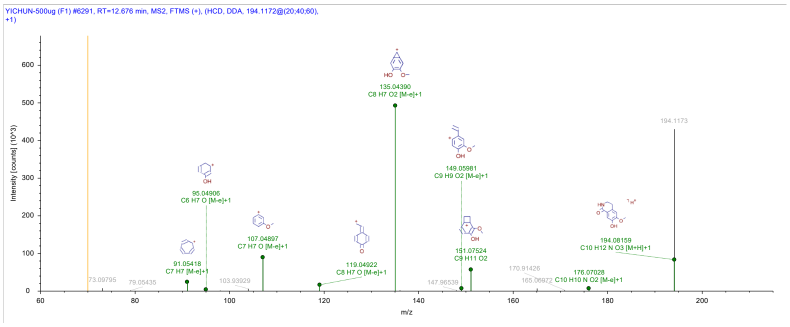

12.866 min 3-hydroxy-2,5-toluqui-none

MS

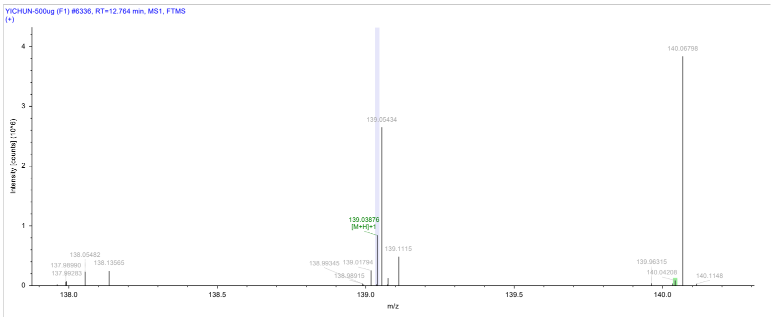

MS/MS

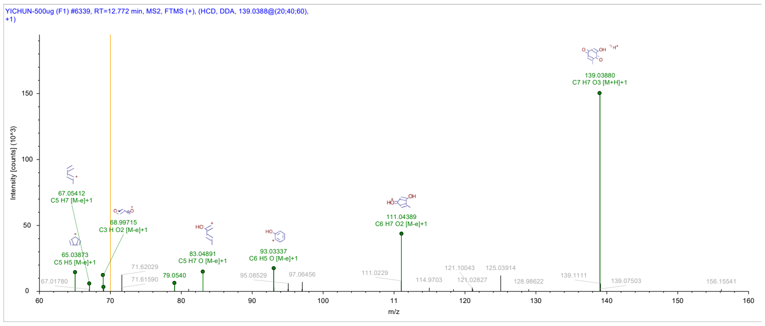

12.99 min *N*-Methylhernovine

MS

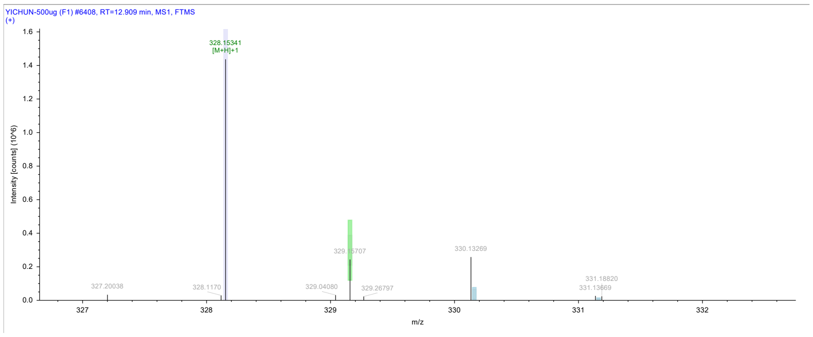

MS/MS

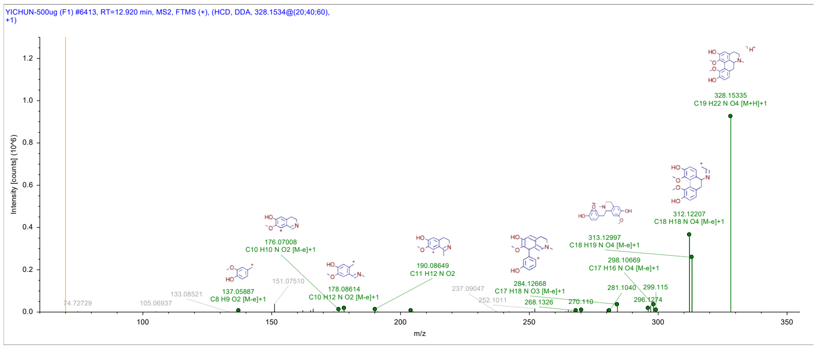

13.008 min Pronuciferine

MS

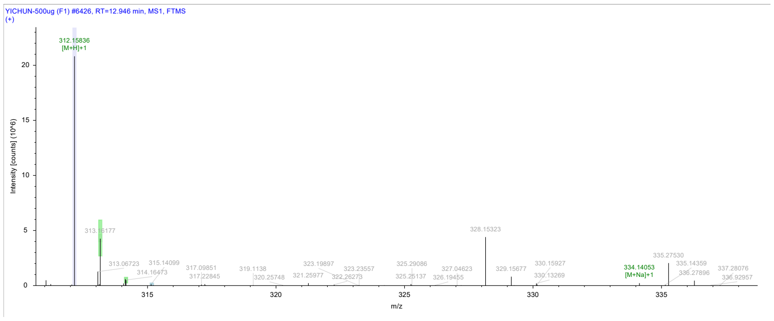

MS/MS

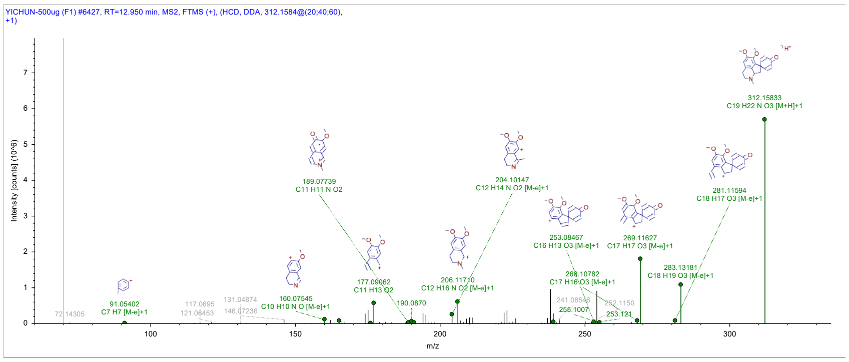

14 min Boldine

MS

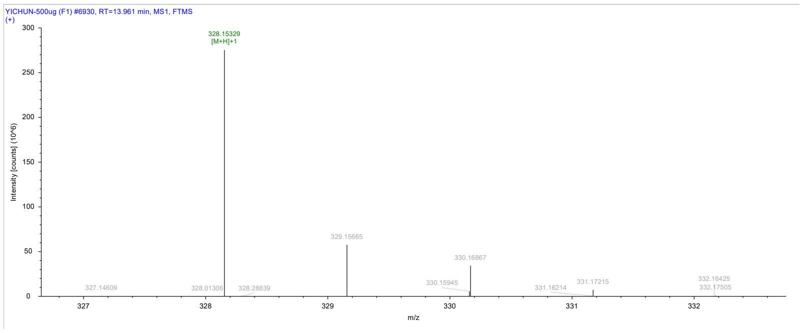

MS/MS

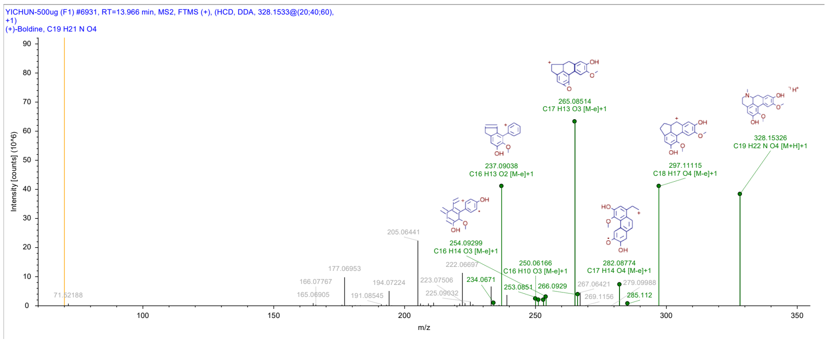

14.138 min Reticuline

MS

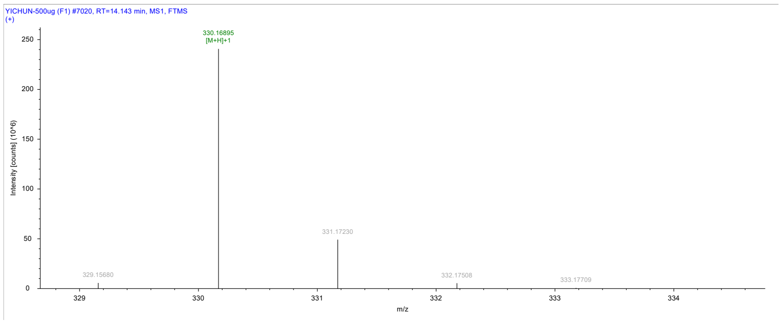

MS/MS

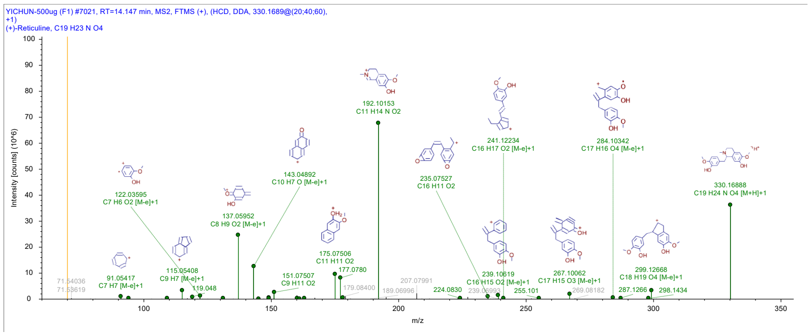

14.242 min Norboldine

MS

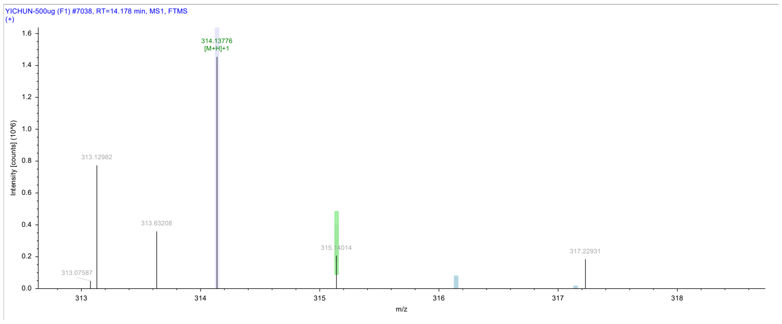

MS/MS

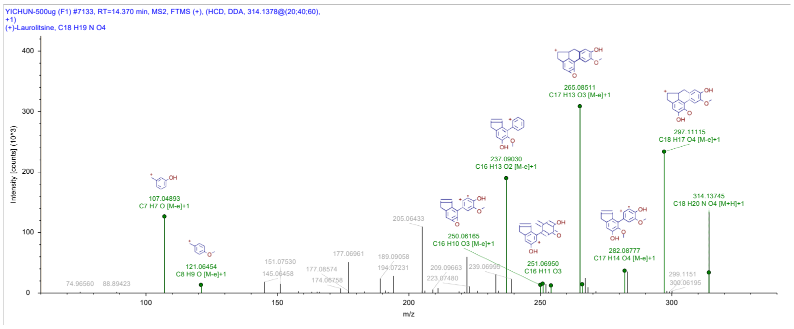

14.818 min 4,6,10,12-Tridecatetraene-2,8-diol

MS

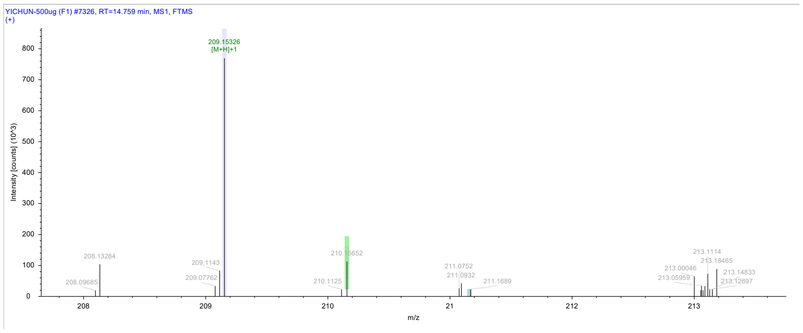

MS/MS

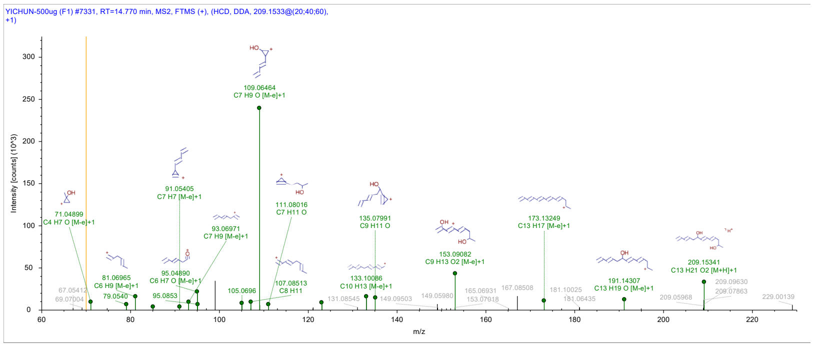

15.924 min Isoboldine

MS

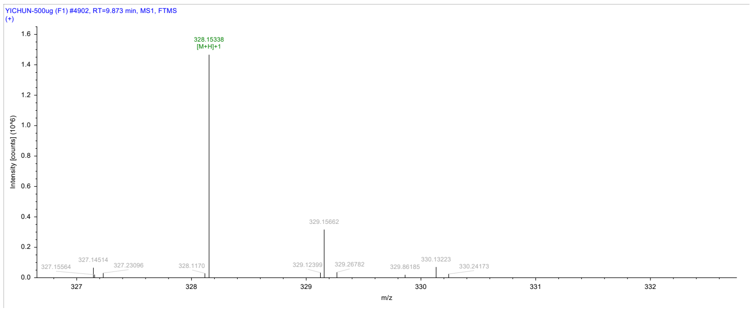

MS/MS

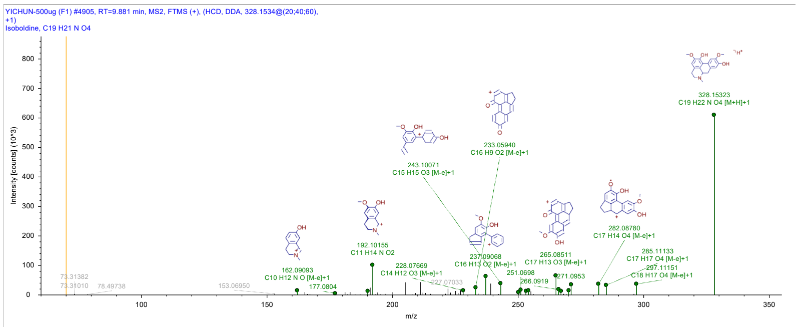

17.327 min 1-(5-oxotetrahydrofuran-2-yl)ethyl 2-phenylacetate

MS

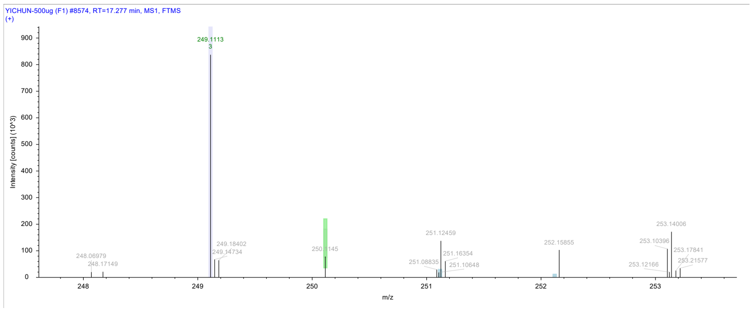

MS/MS

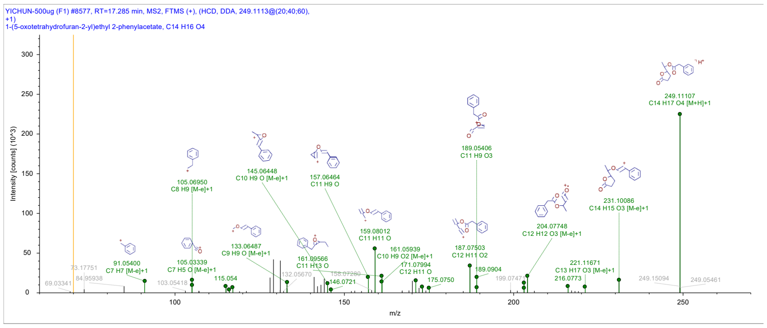

17.398 min Isocorydine

MS

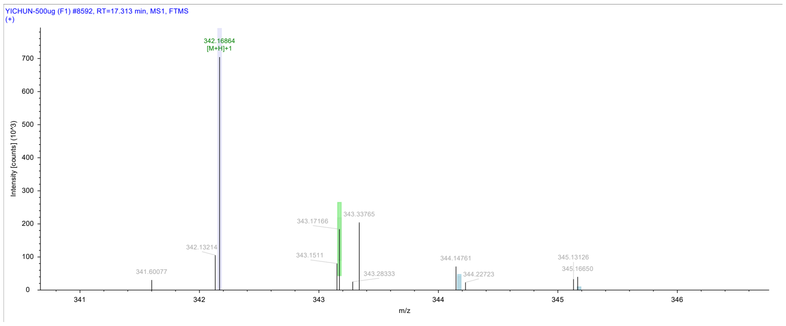

MS/MS

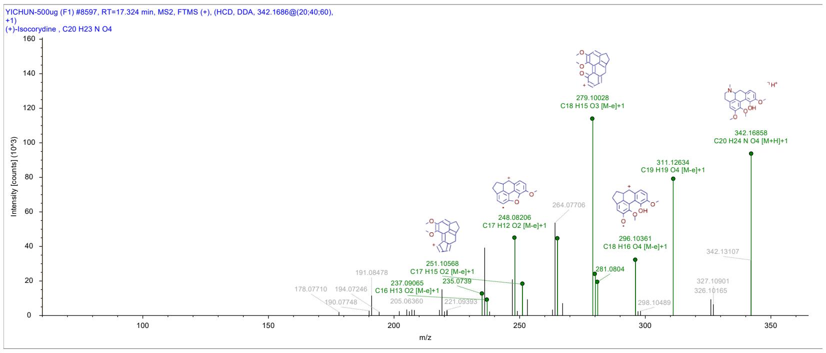

18.013 min Epipyriculol

MS

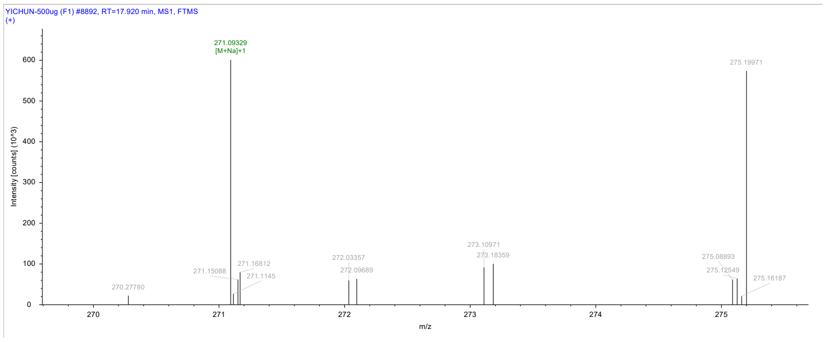

MS/MS

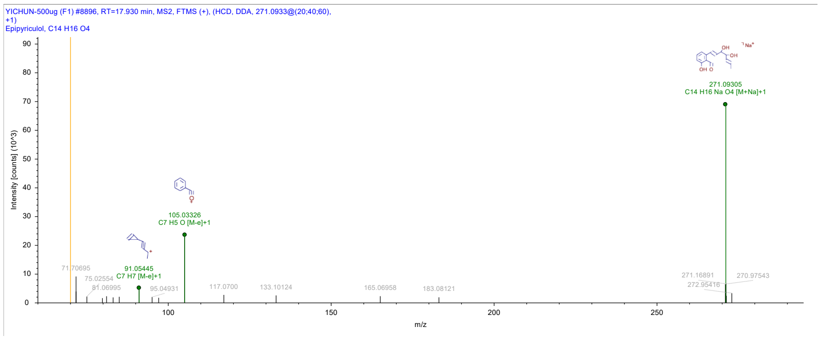

18.58 min *N*-Methyllaurotetanine

MS

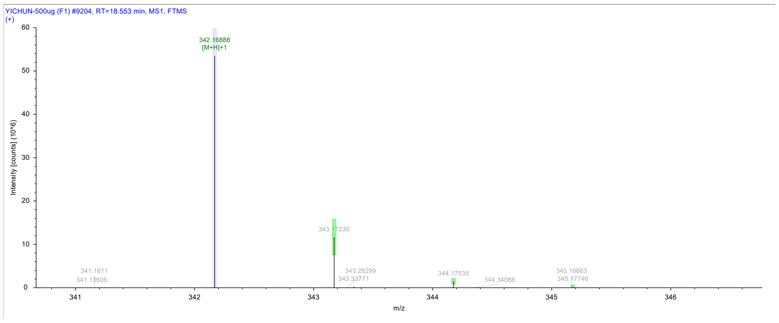

MS/MS

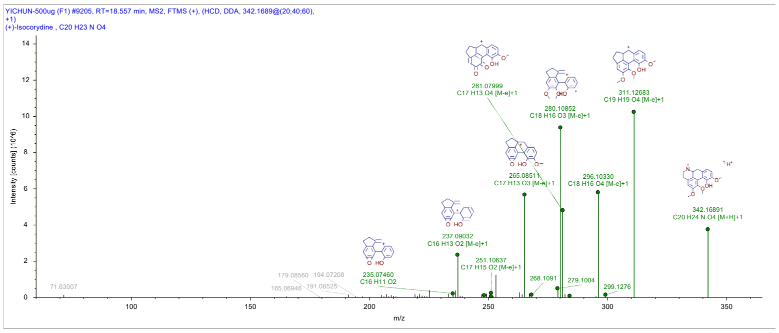

18.915 min Linderolide S

MS

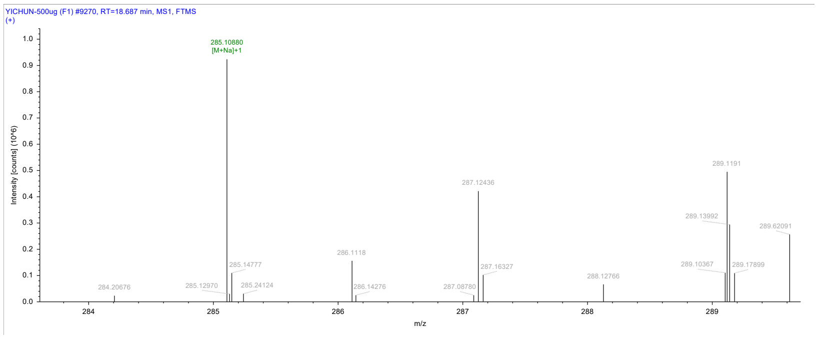

MS/MS

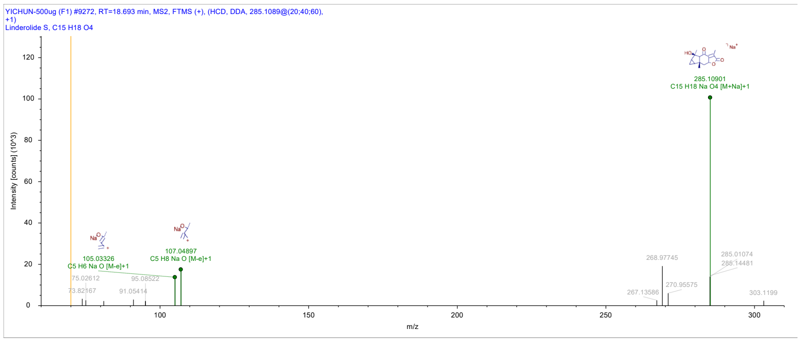

20.637 min 9-Hydroxy-1,2,10-trimethoxy-7H-dibenzo[de,g]quinolin-7-one

MS

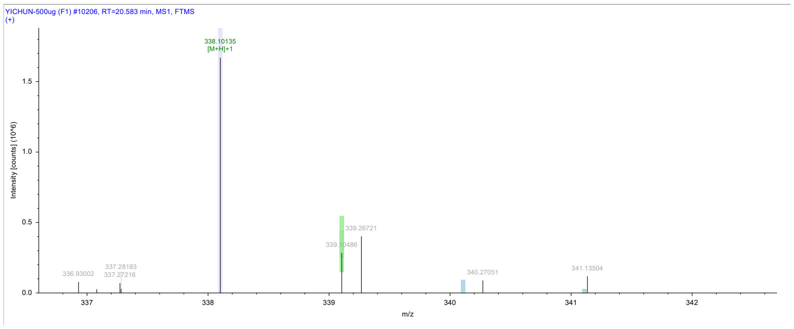

MS/MS

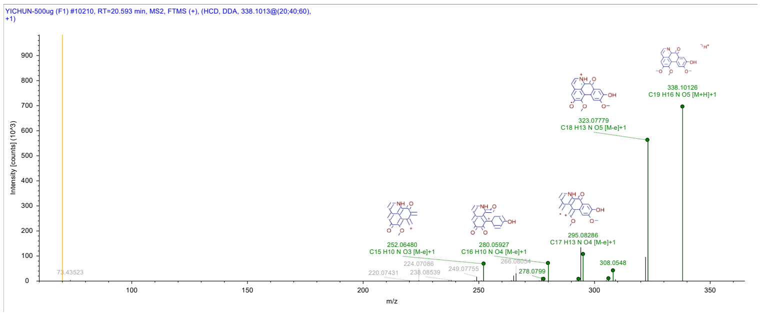

20.757 min Karakoramine

MS

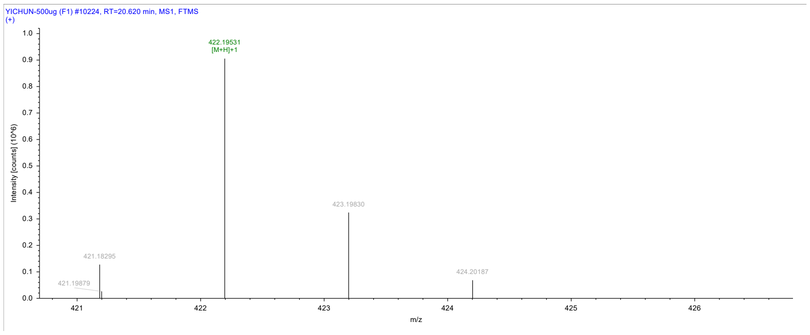

MS/MS

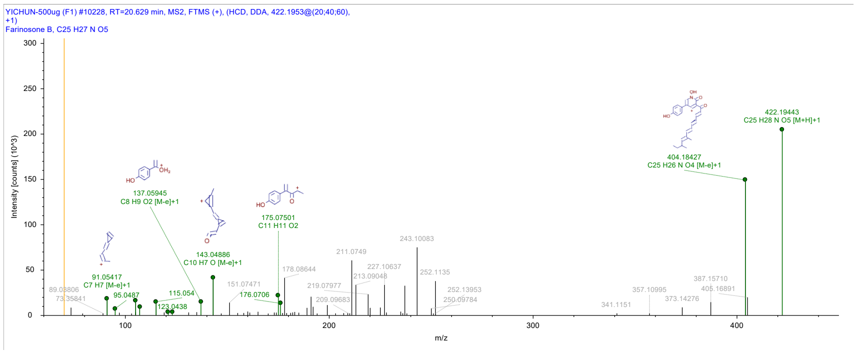

20.807 min Norarmepavine

MS

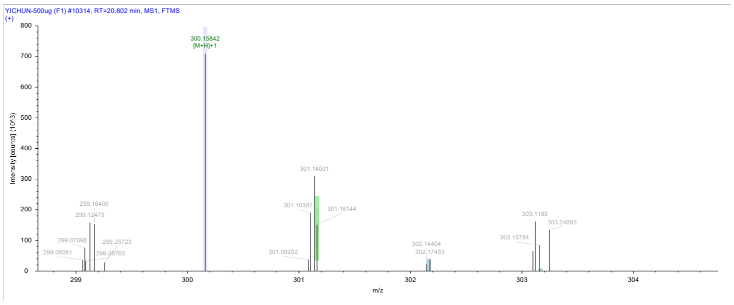

MS/MS

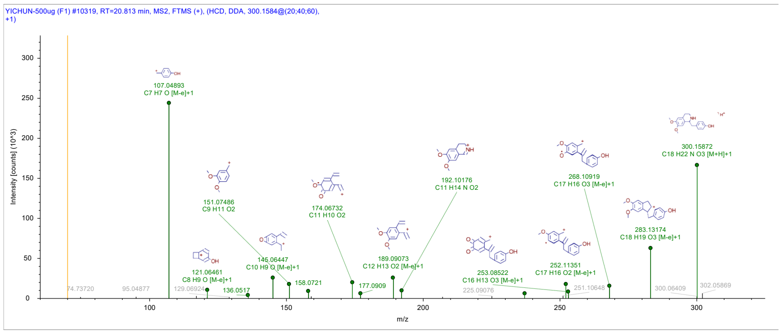

20.884 min Linderolide D

MS

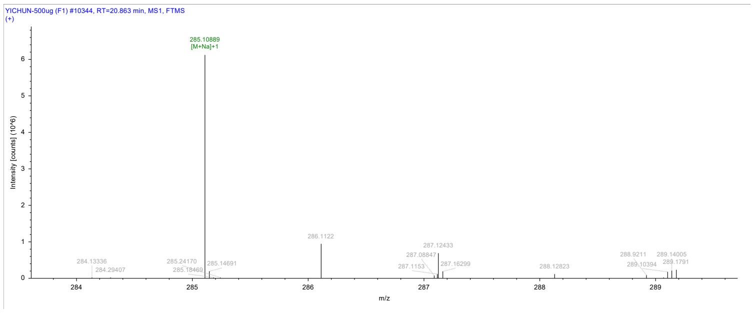

MS/MS

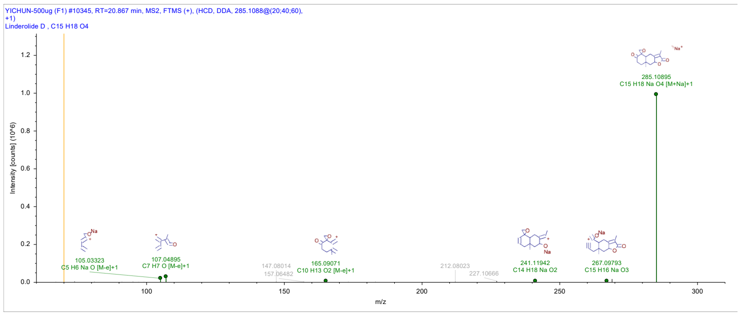

20.89 min Neolinderalactone

MS

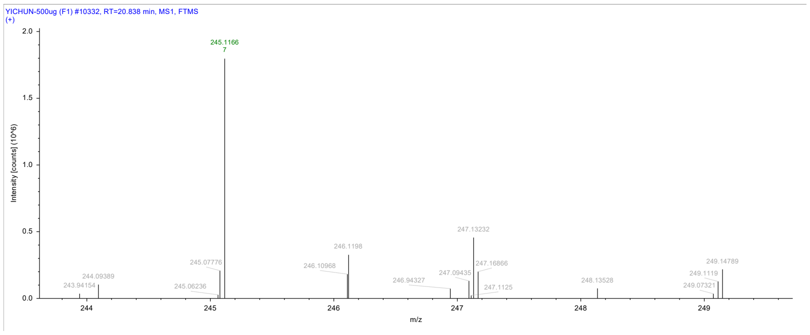

MS/MS

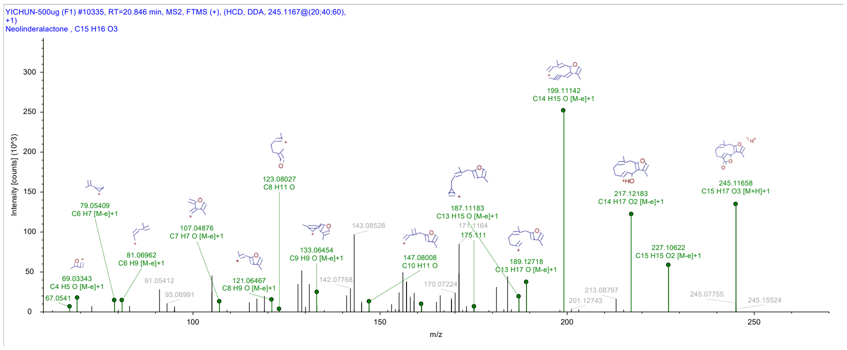

21.927 min *N*-Feruloyltyramine

MS

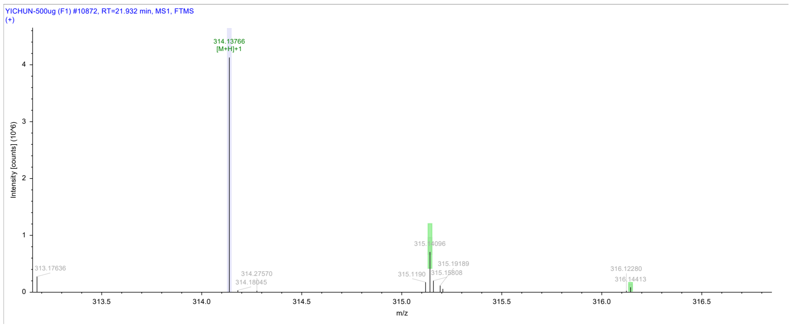

MS/MS

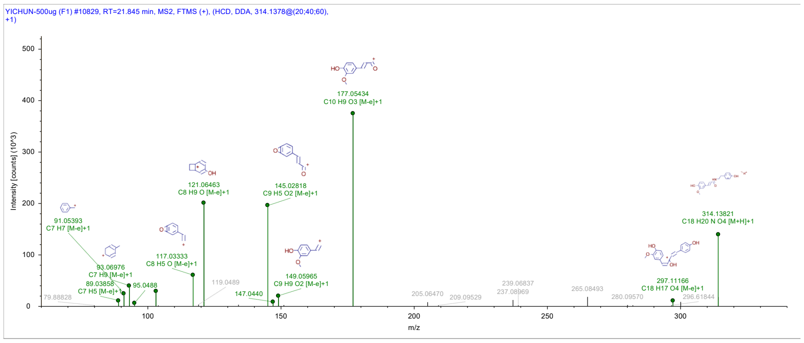

22.125 min Linderolide B

MS

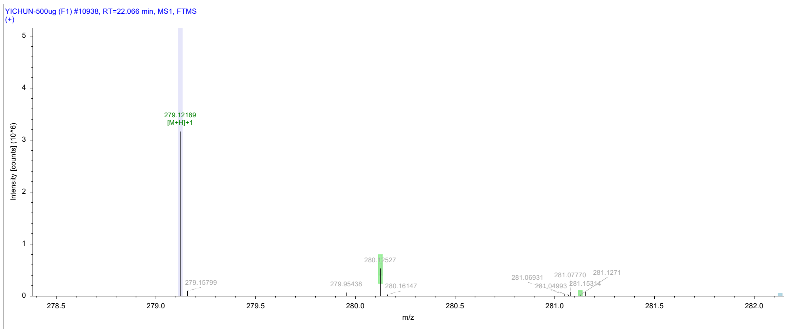

MS/MS

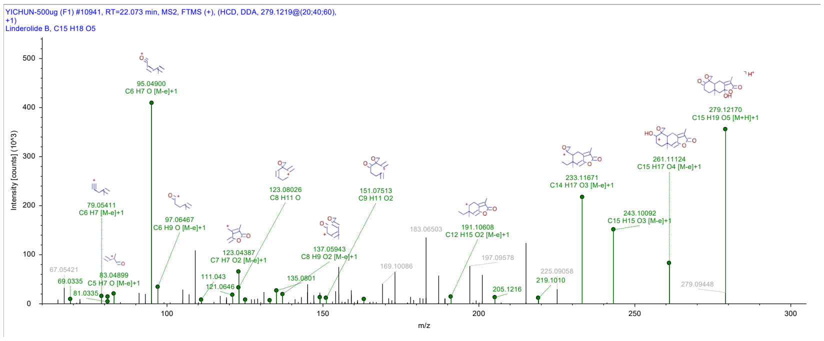

22.901 min Peniophoral

MS

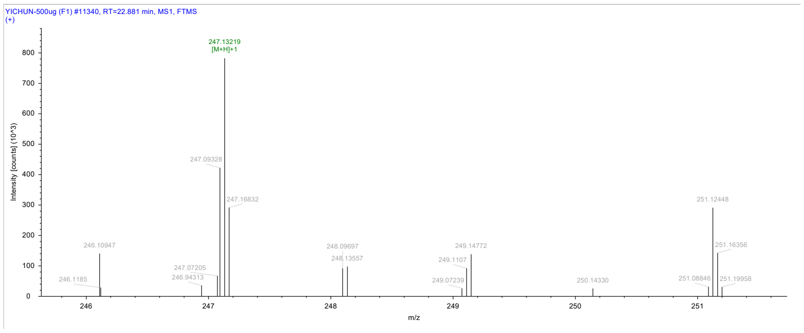

MS/MS

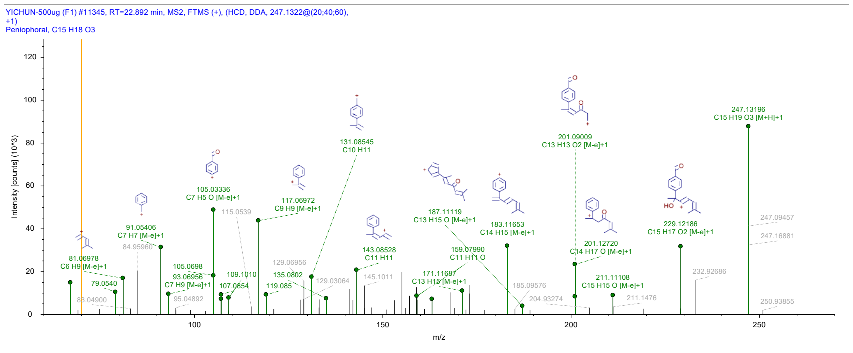

24.674 min 8-Hydroxylindestenolide

MS

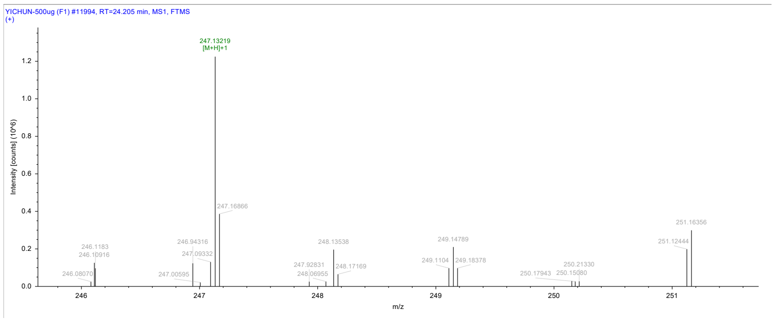

MS/MS

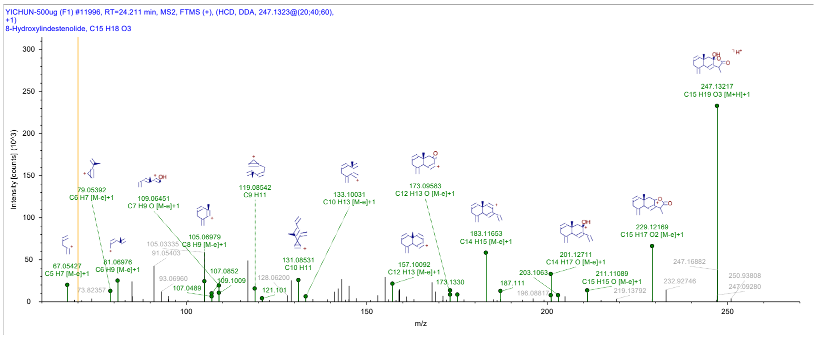

25.094 min Parvigemone

MS

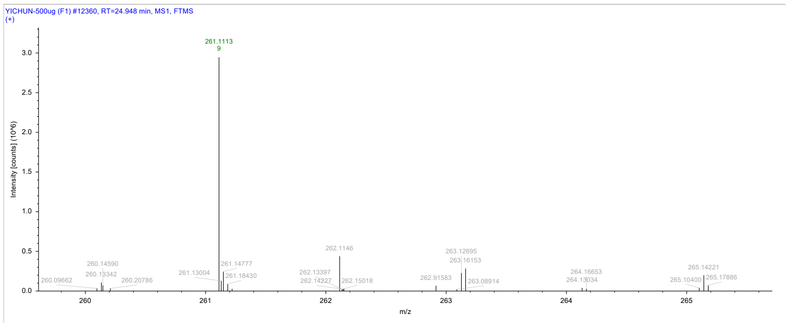

MS/MS

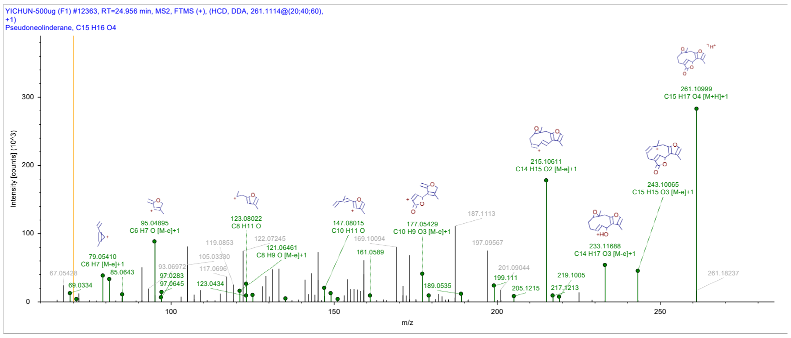

25.788 min Citreoviripyrone B

MS

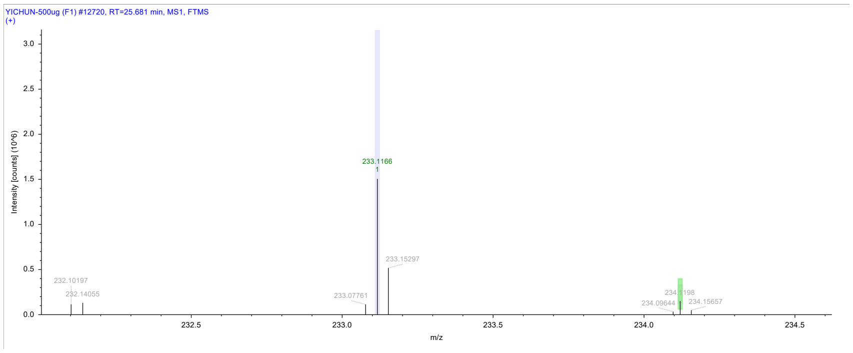

MS/MS

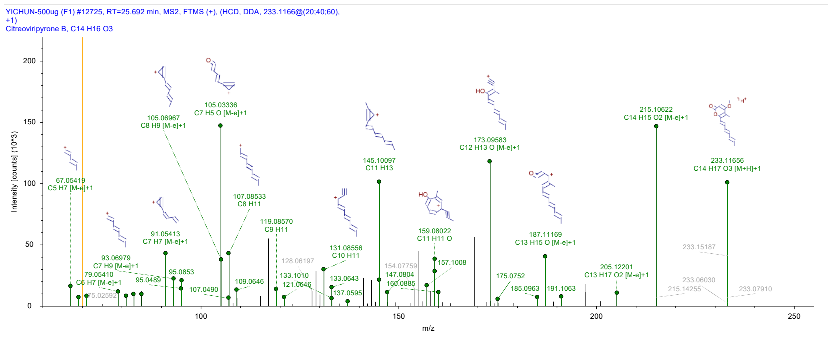

26.105 min Linderolide I

MS

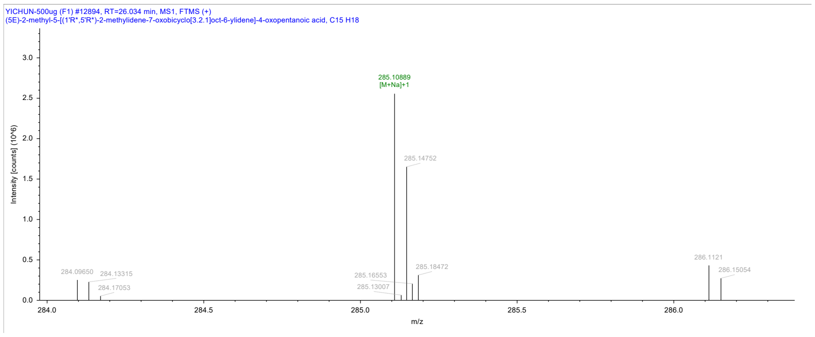

MS/MS

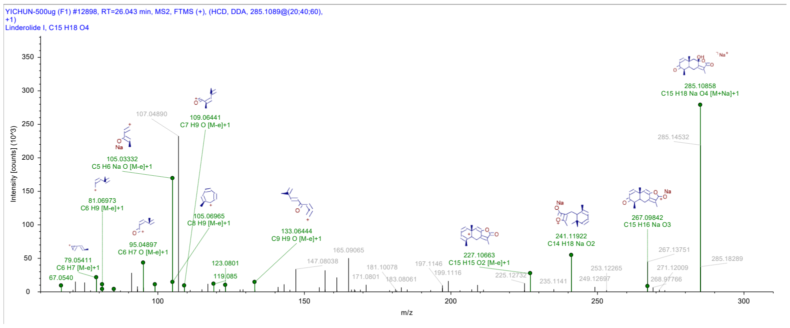

26.402 min 1-methylabscisic-6-acid

MS

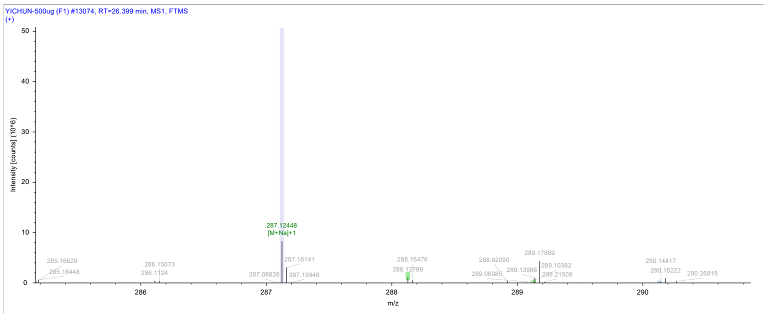

MS/MS

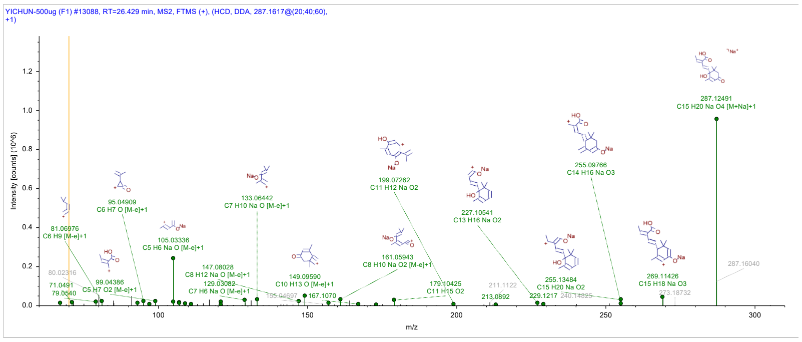

26.419 min 2-methyl butyl propyl phthalate

MS

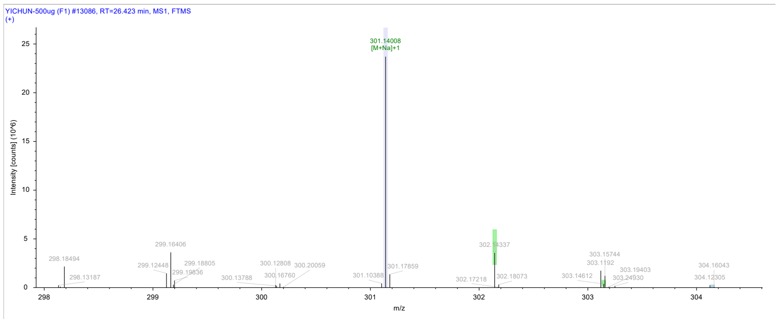

MS/MS

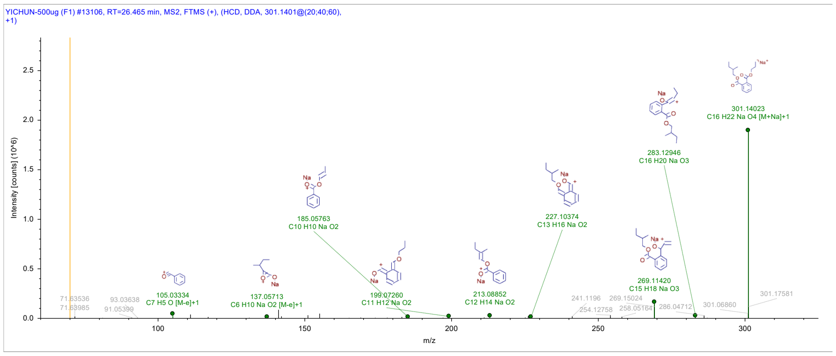

26.622 min β-Ionone

MS

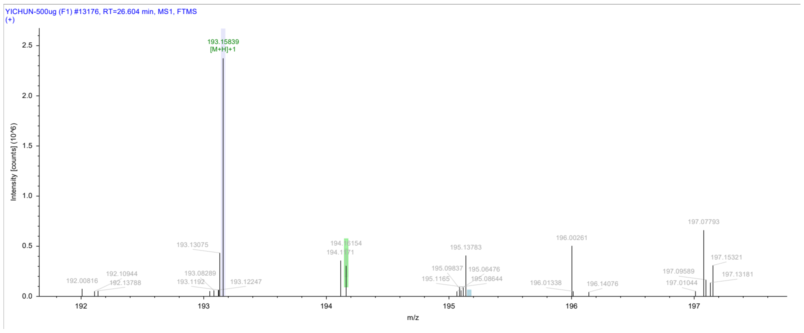

MS/MS

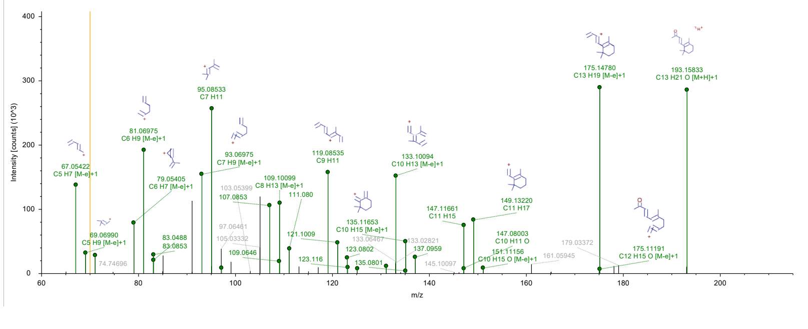

26.727 min Dehydrocostus lactone

MS

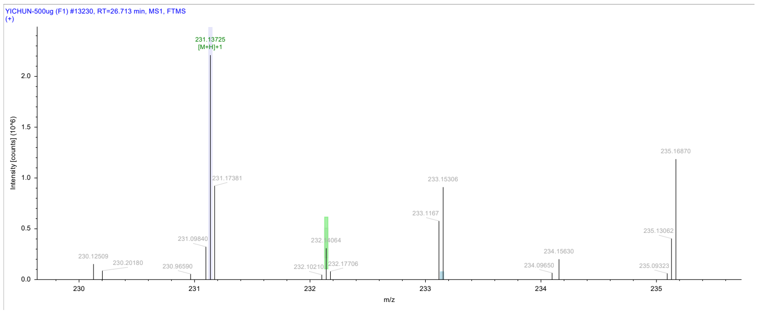

MS/MS

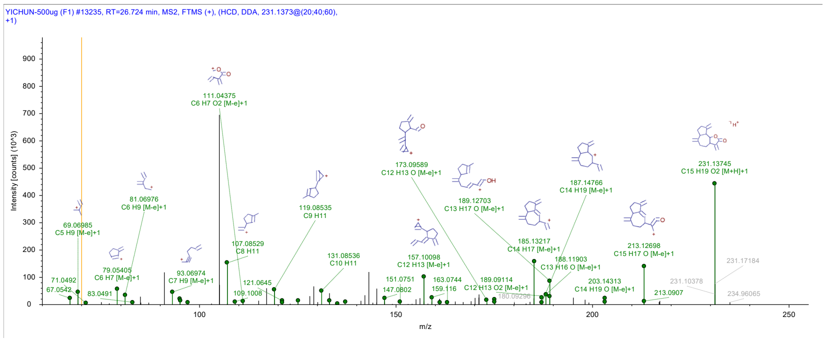

27.194 min Lactarioline A

MS

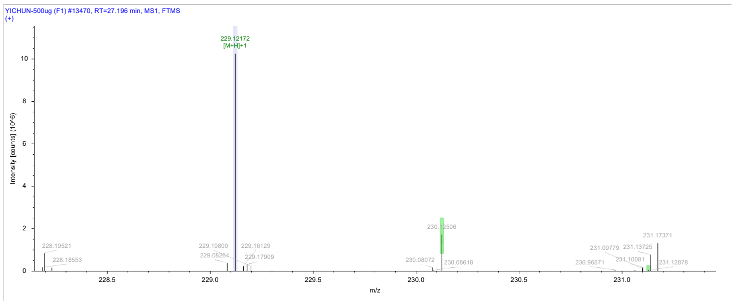

MS/MS

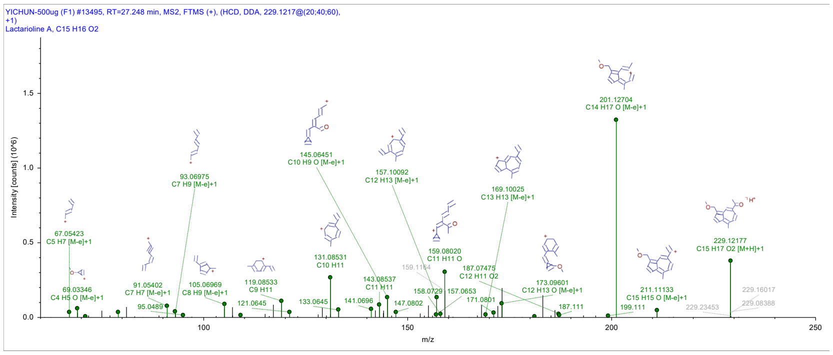

27.639 min α -Farnesene

MS

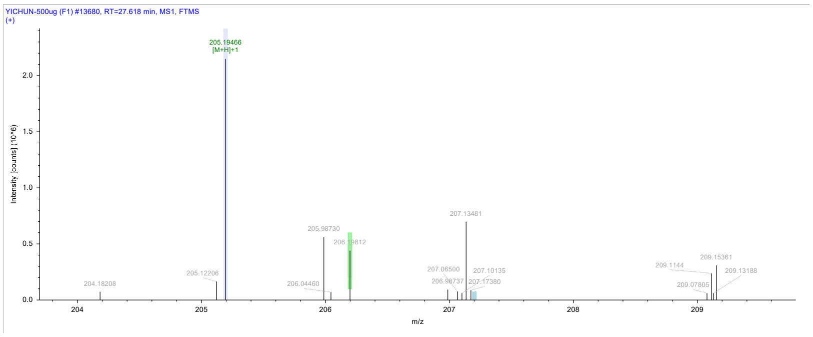

MS/MS

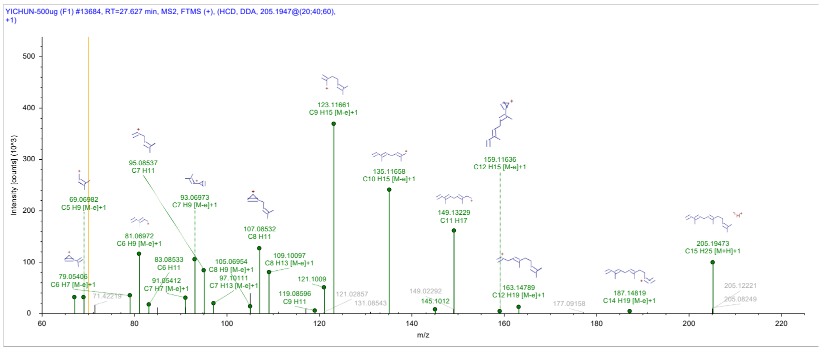

27.999 min Lindestrenolide

MS

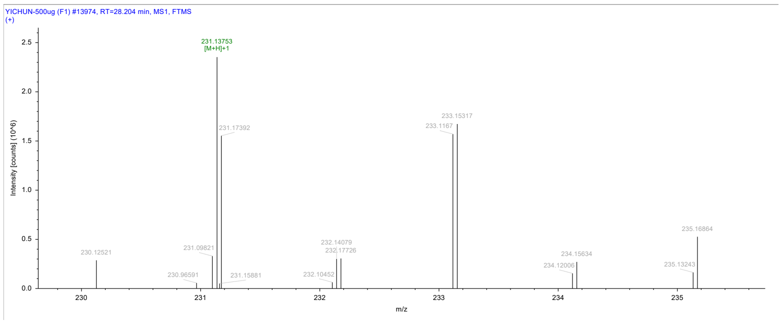

MS/MS

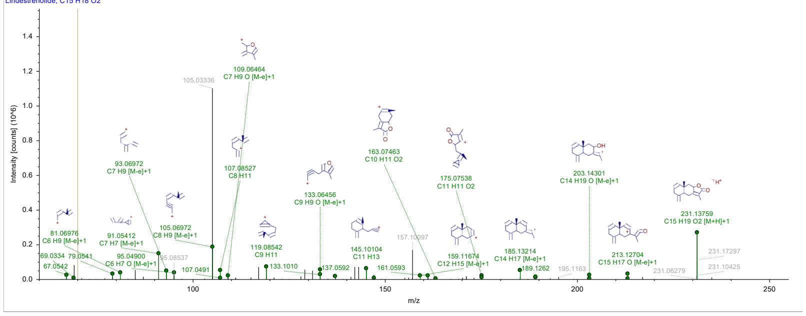

28.054 min Calamenene

MS

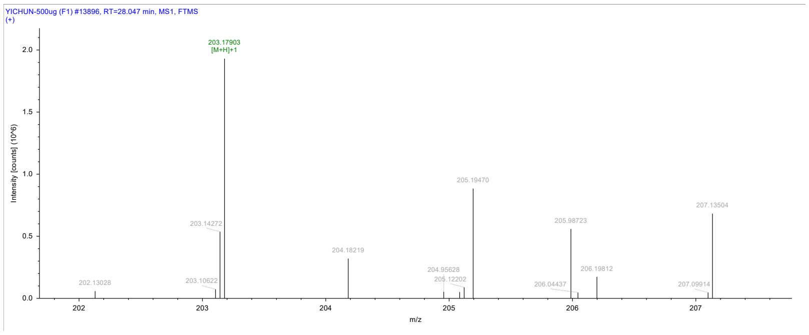

MS/MS

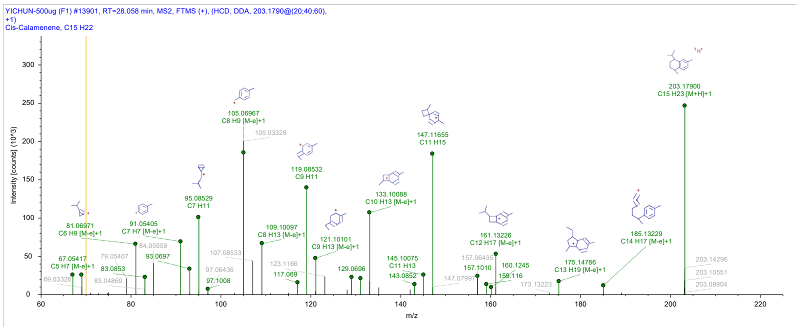

28.159 min 1-Phenylhept-3-en-4-one

MS

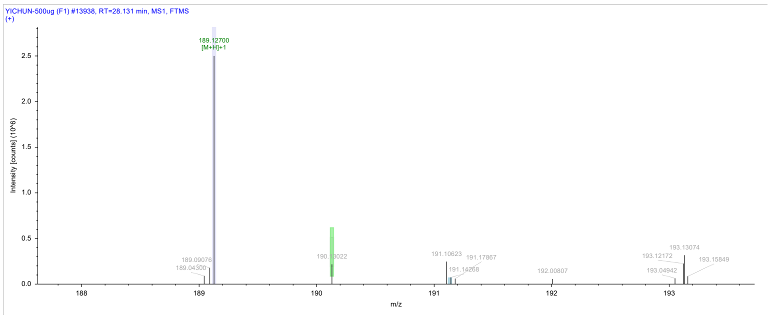

MS/MS

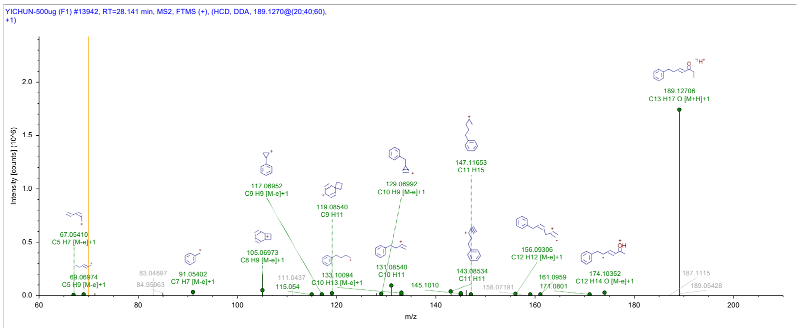

28.288 min 2,4,6,8,10,12-tetradeca-1,14-hexenedial

MS

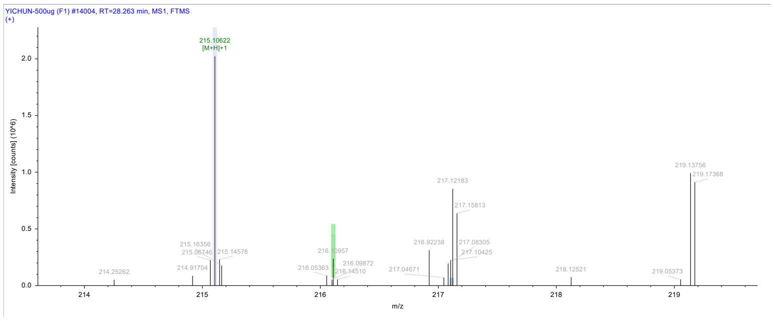

MS/MS

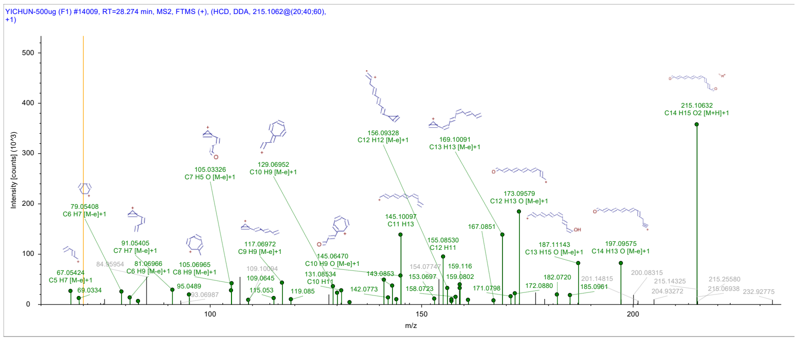

28.299 min Linderane

MS

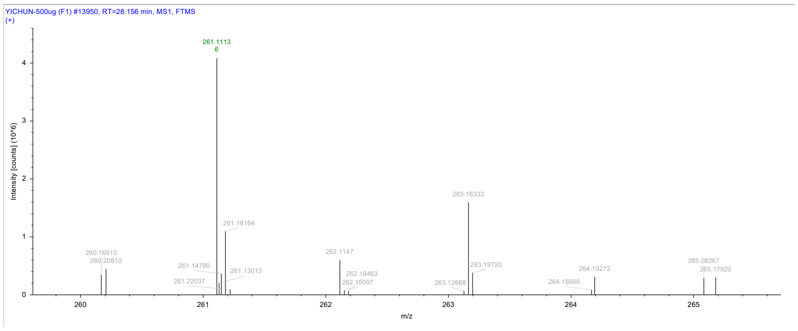

MS/MS

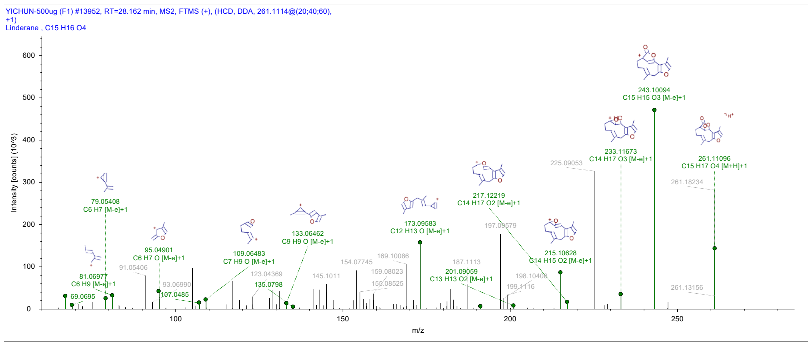

28.448 min 10-Methyldodec-3-en-4-olide

MS

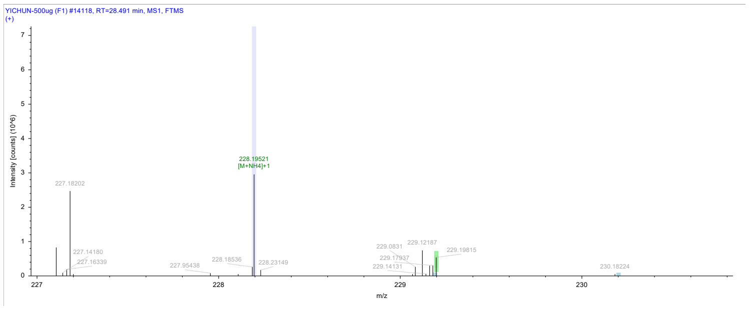

MS/MS

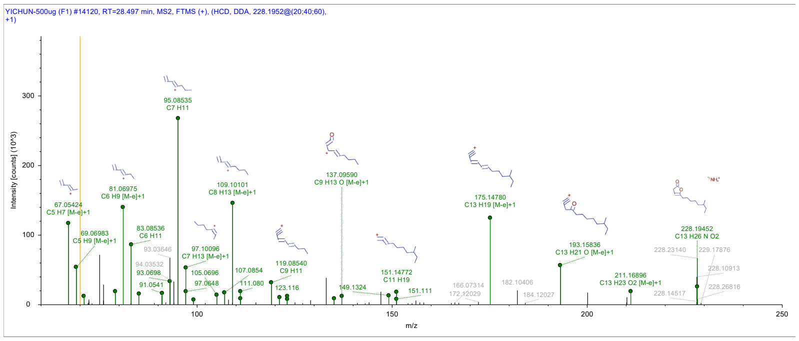

28.695 min Isolinderalactone

MS

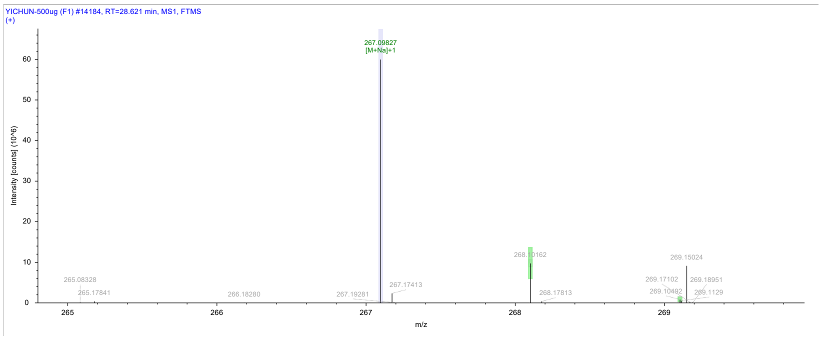

MS/MS

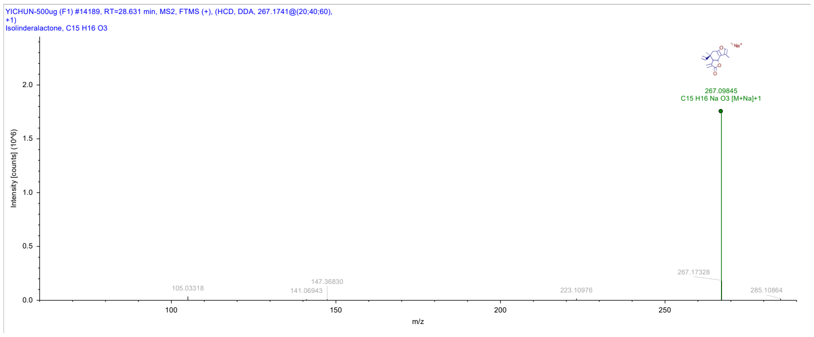

28.742 min Lindenenol

MS

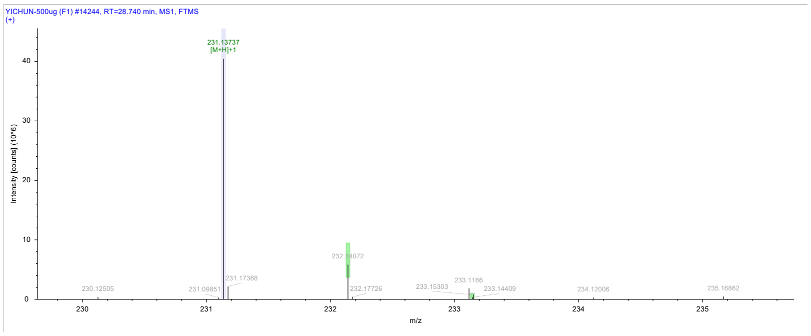

MS/MS

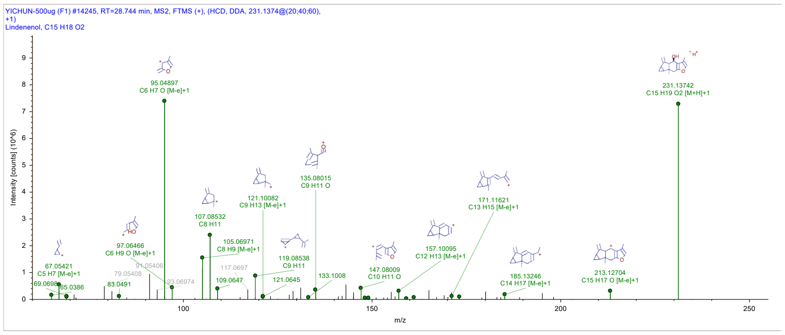

28.74 min Linderoxide

MS

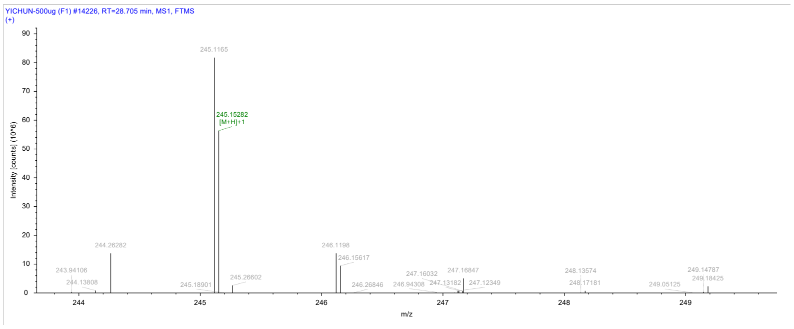

MS/MS

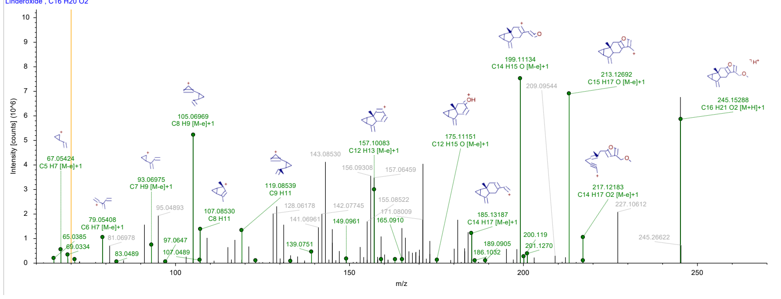

28.764 min Benquoine

MS

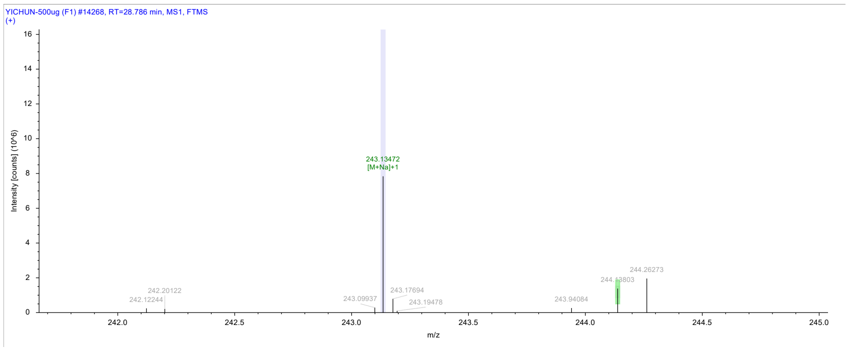

MS/MS

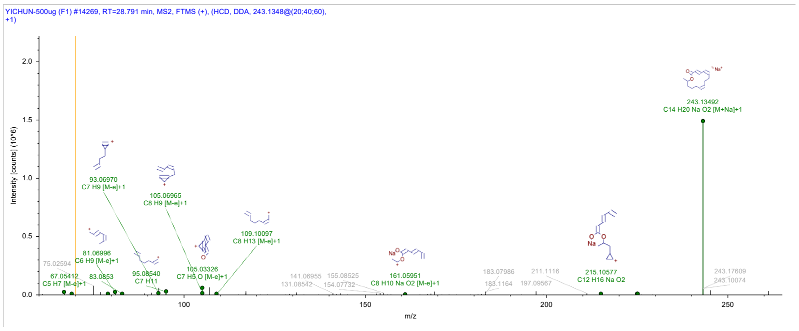

28.807min Anaephene A

MS

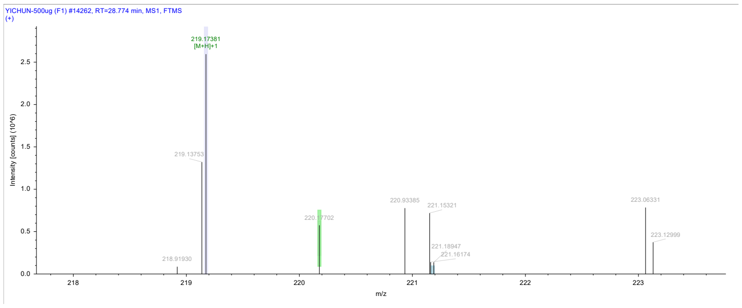

MS/MS

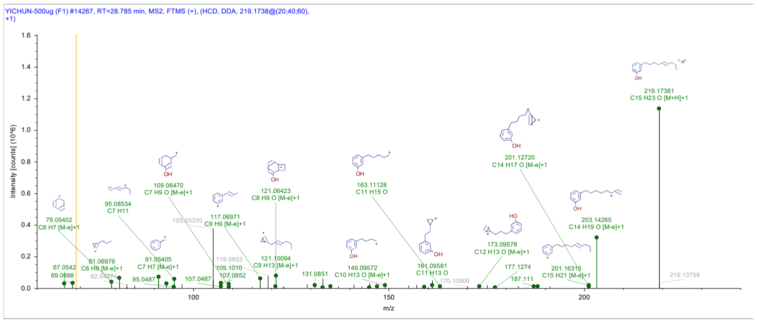

28.957 min Dehydrolindestrenolide

MS

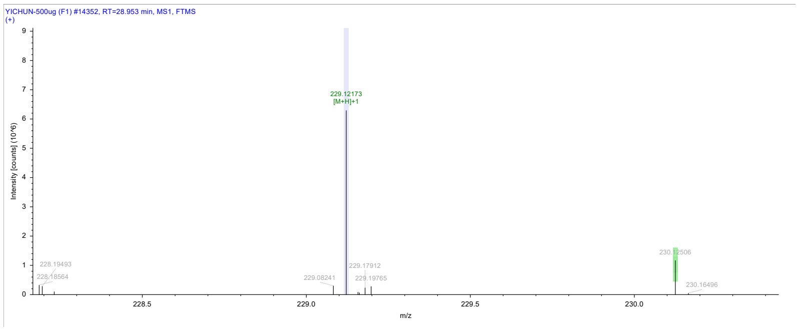

MS/MS

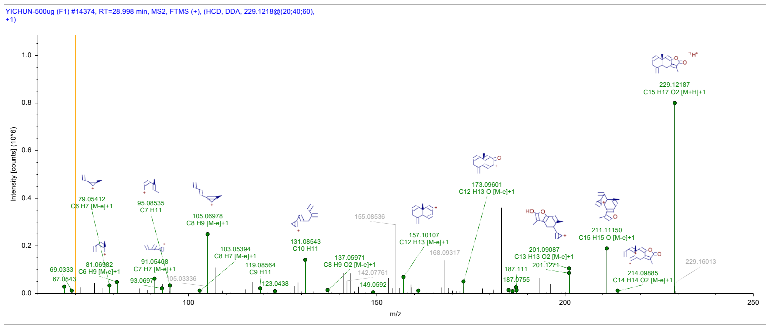

29.195 min Lindenanolide A

MS

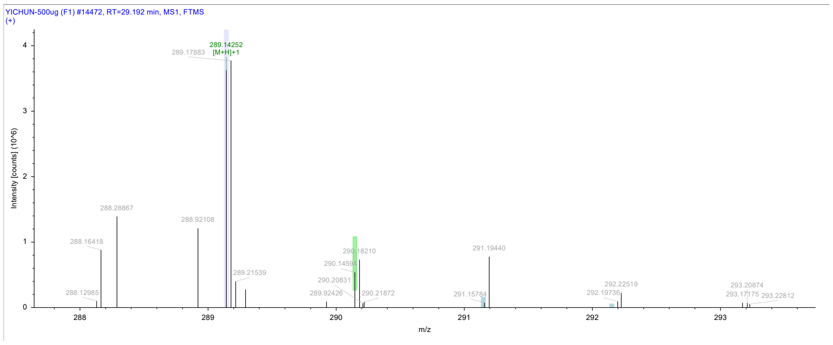

MS/MS

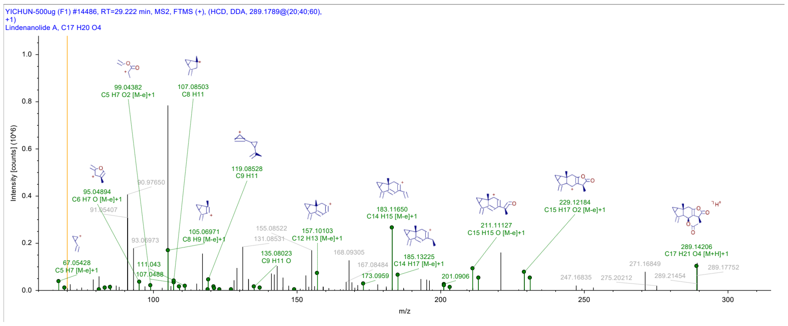

29.58 min Nigriterpene E

MS

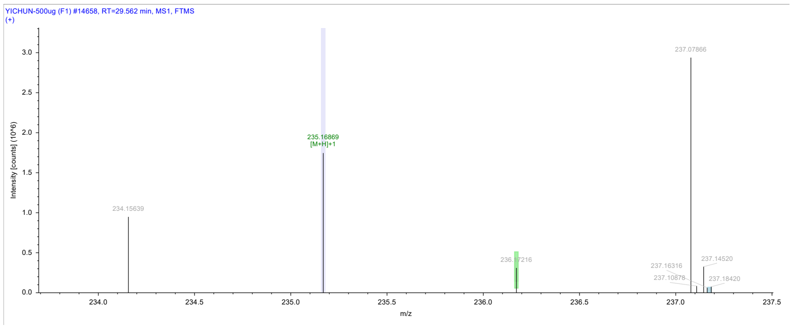

MS/MS

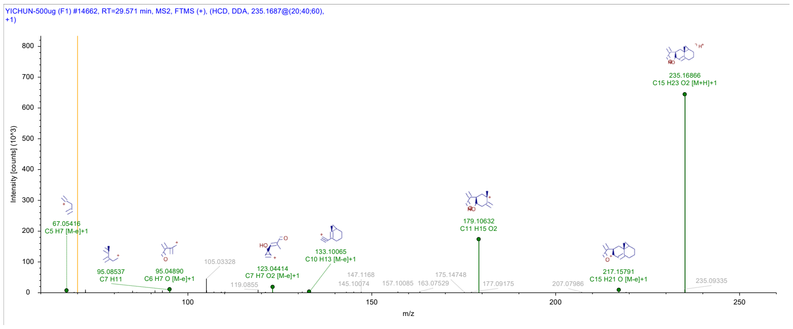

30.521 min Shizukanolide A

MS

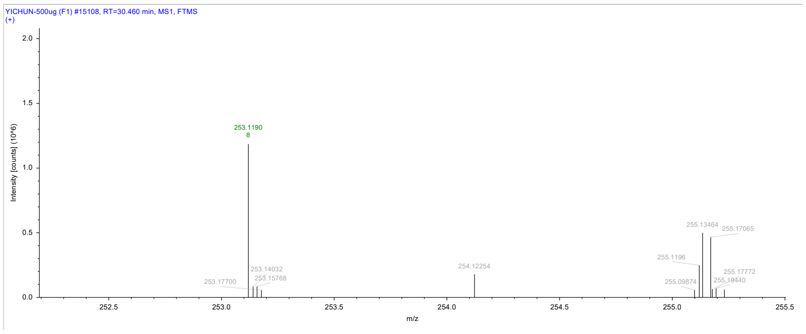

MS/MS

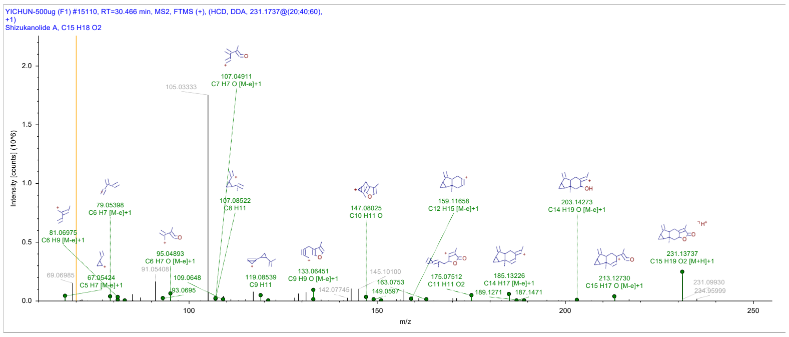

30.57 min Rulepidadiene B

MS

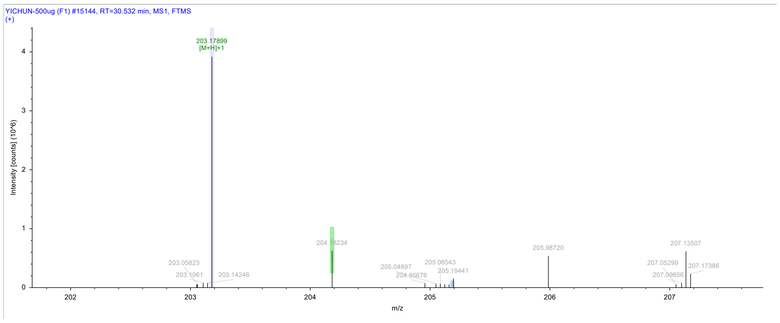

MS/MS

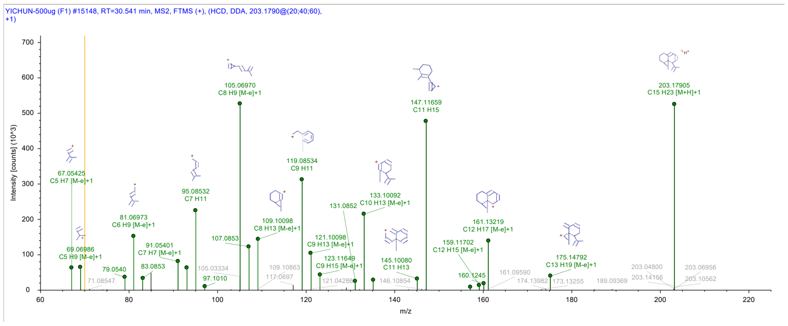

33.03 min Linderone

MS

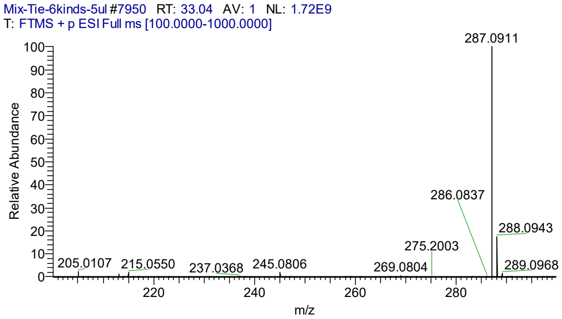

MS/MS

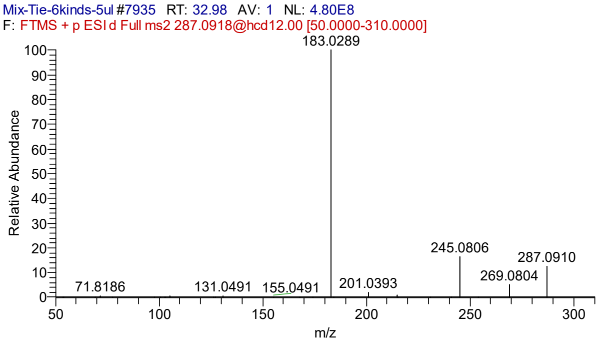

31.272 min Lorneic acid J

MS

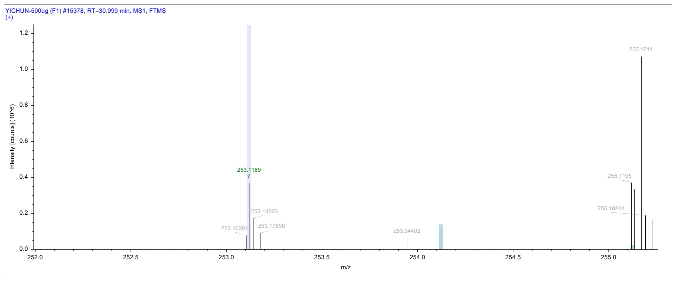

MS/MS

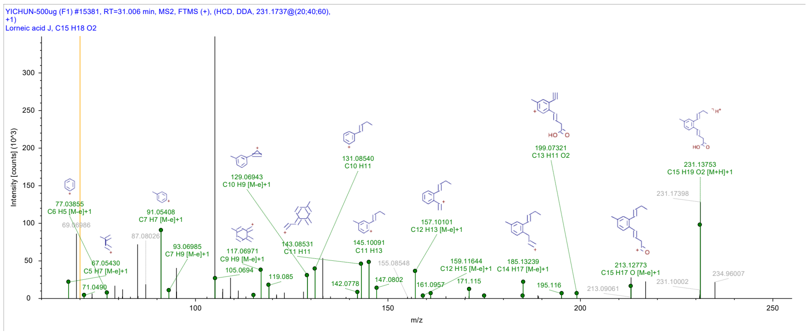

31.299 min (+)-α-Cyperone

MS

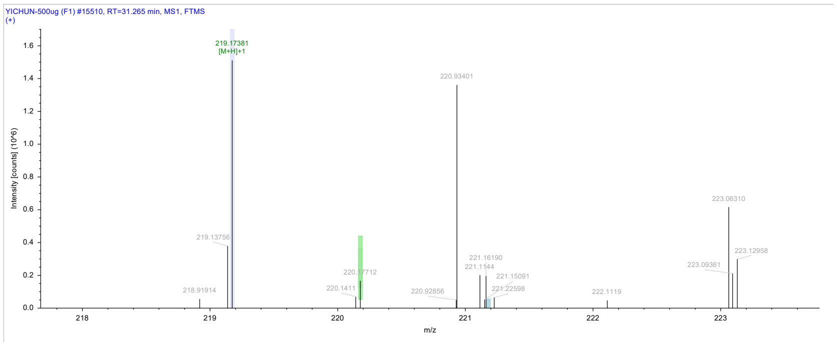

MS/MS

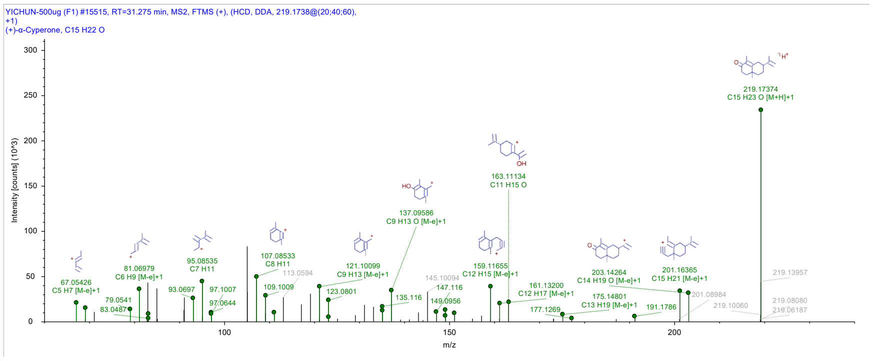

32.087 min Lindereneyl acetate

MS

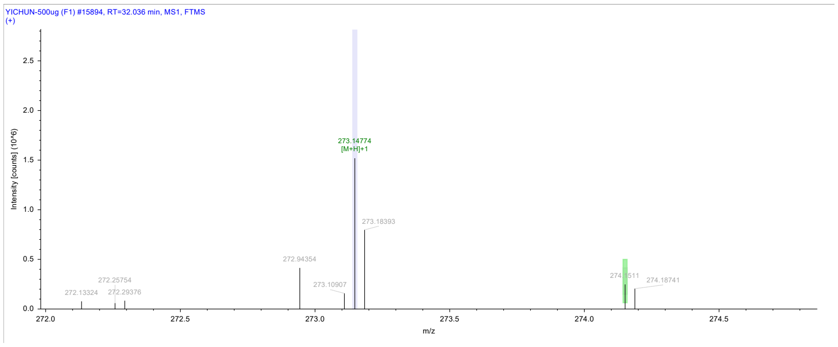

MS/MS

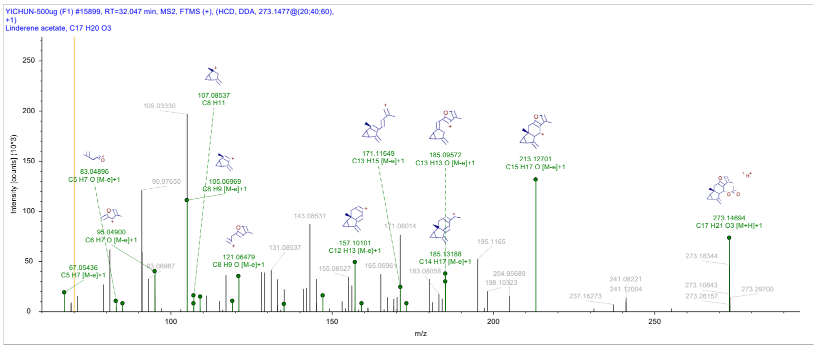

32.093 min Isolinderoxide

MS

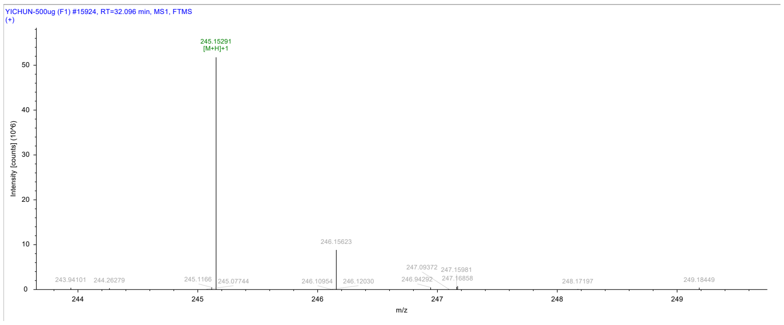

MS/MS

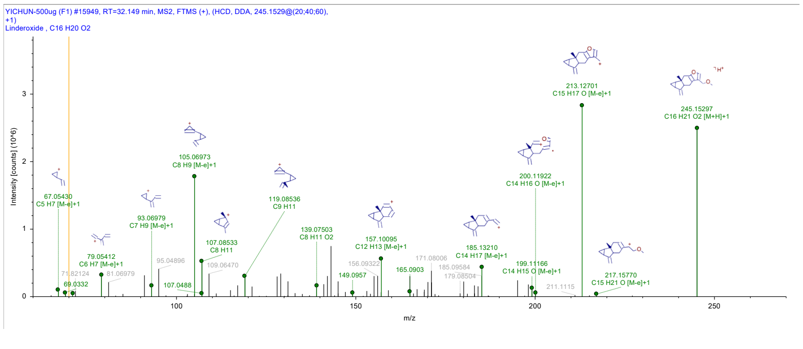

32.92 min (2*E*,3*R*,4*S*)-2-dodecylinene-3-hydroxy-4-ethoxy-4-methylbutanolide

MS

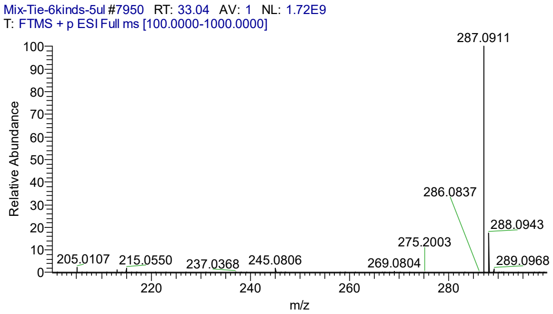

MS/MS

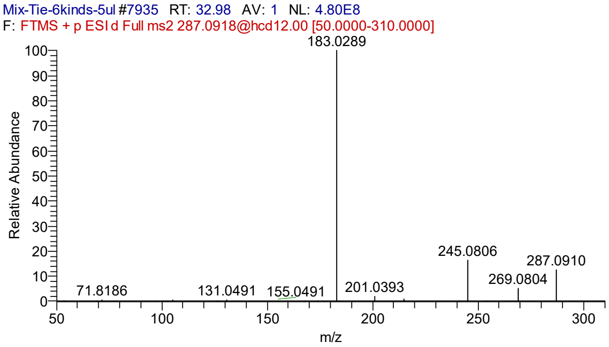

32.966 min (4aS,8aS)-4,4a,5,6,8a,9-Hexahydro-3,8a-dimethyl-5-methylenenaphtho[2,3-b]furan

MS

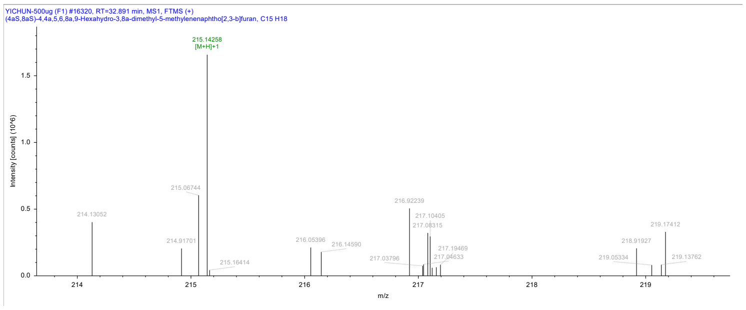

MS/MS

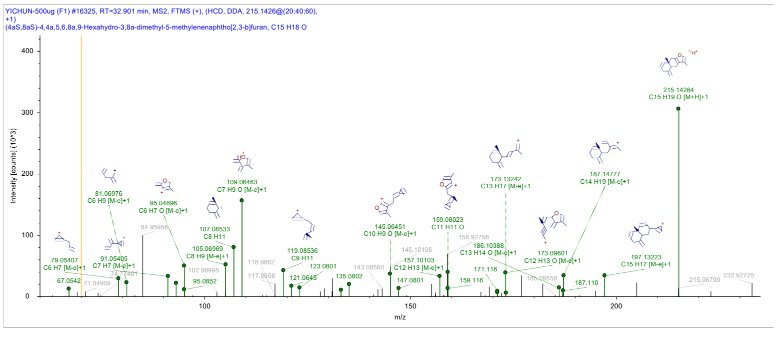

32.99 min 1-acetyl-4-methoxyl-denudaquinol

MS

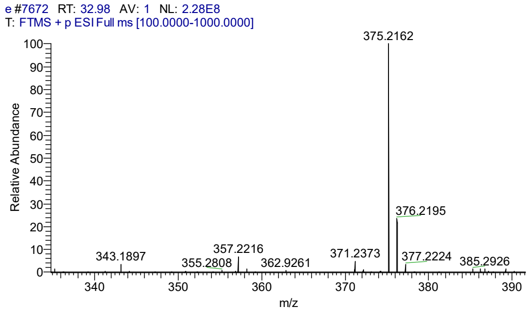

MS/MS

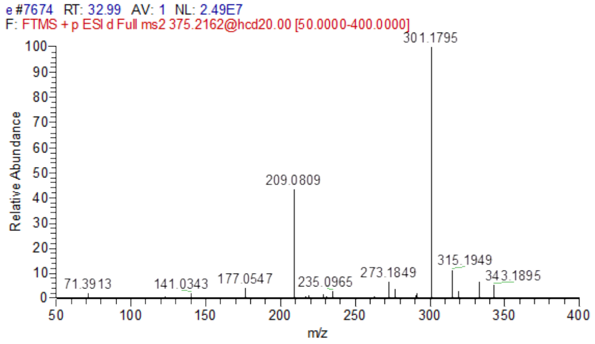

33.03 min methyllinderone

MS

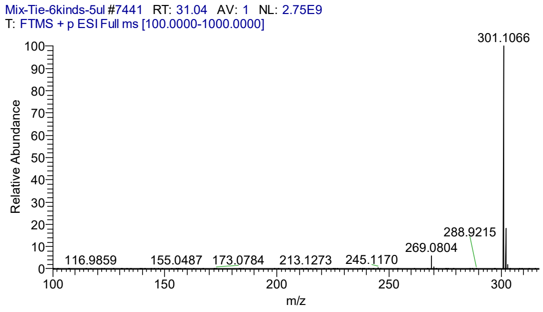

MS/MS

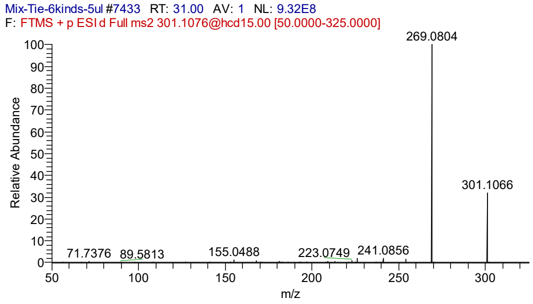

33.29 min linderin B

MS

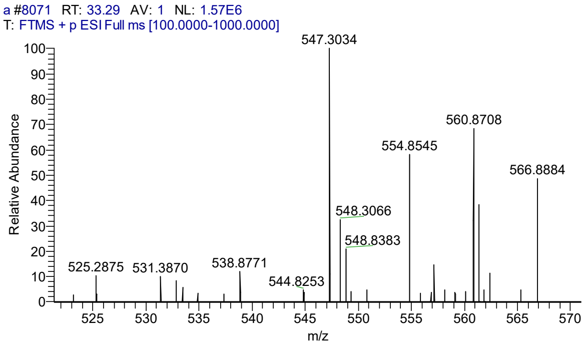

MS/MS

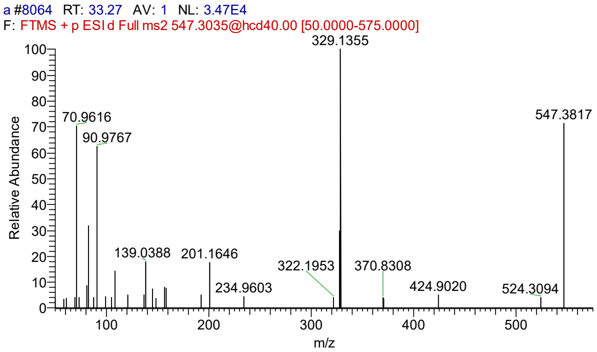

33.634 min Hexadecanamide

MS

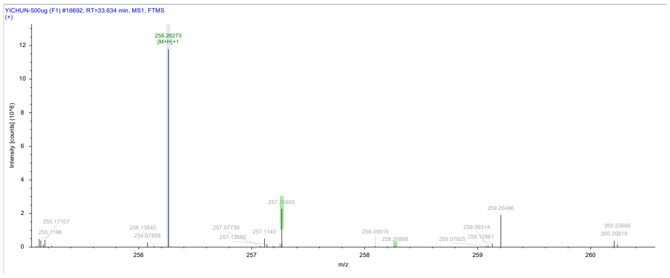

MS/MS

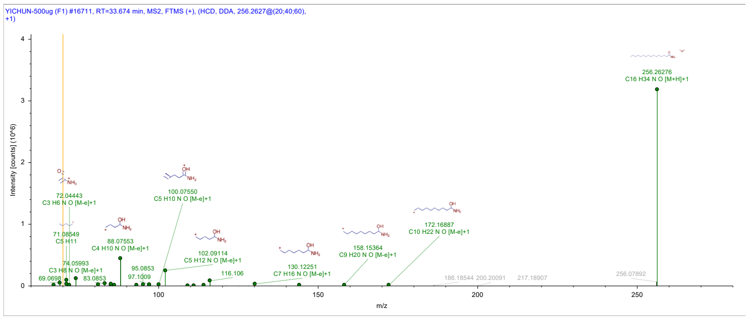

34.49 min (2*E*,3*R*,4*S*)-2-tetradecylinene-3-hydroxy-4-ethoxy-4-methylbutanolide

MS

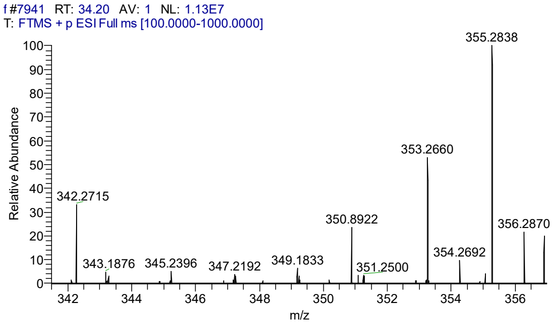

MS/MS

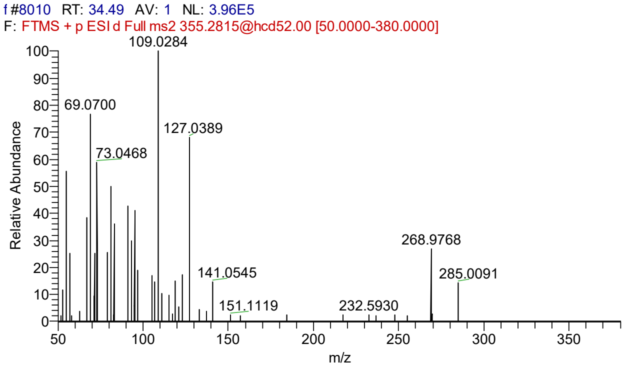

34.558 min Coniferyl aldehyde

MS

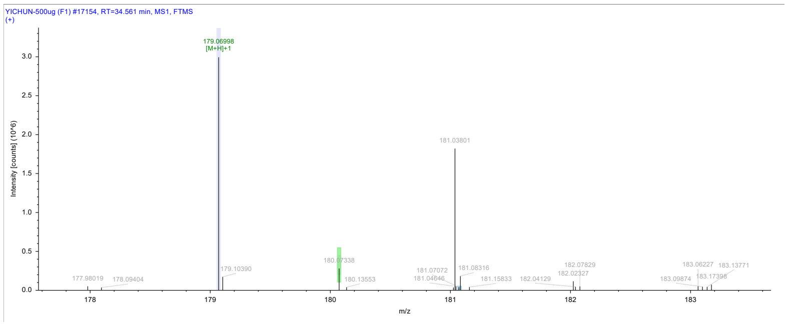

MS/MS

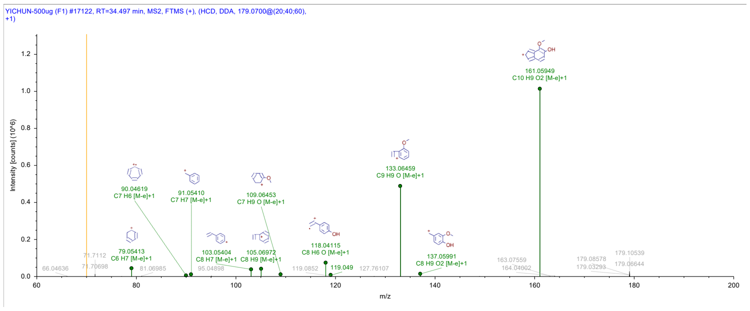

34.559 min Ralfuranone A

MS

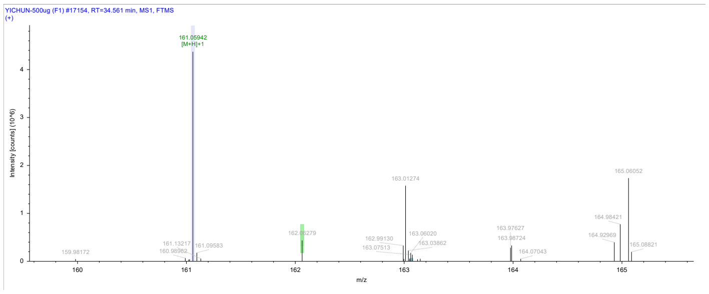

MS/MS

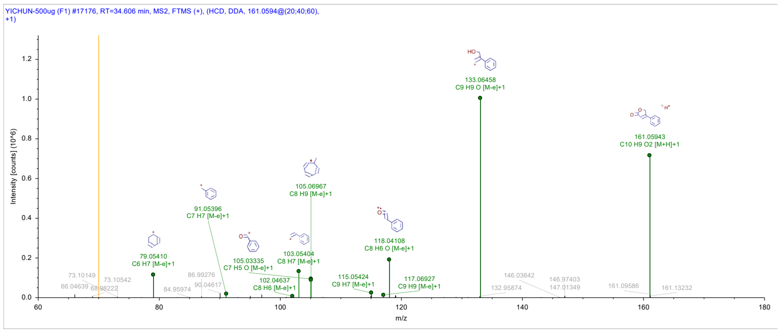

34.83 min lindechunisin A

MS

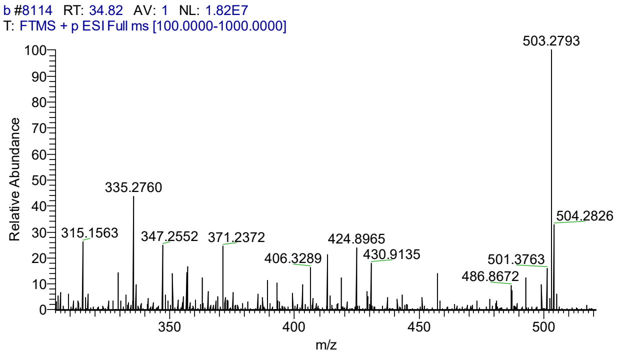

MS/MS

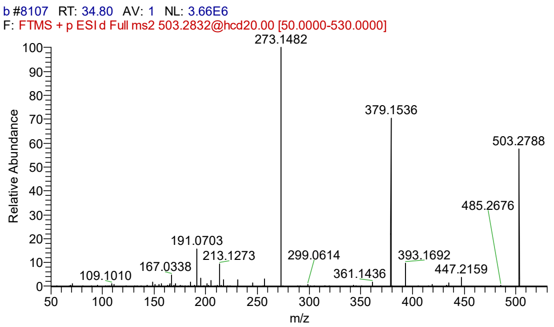

35.129 min Erucamide

MS

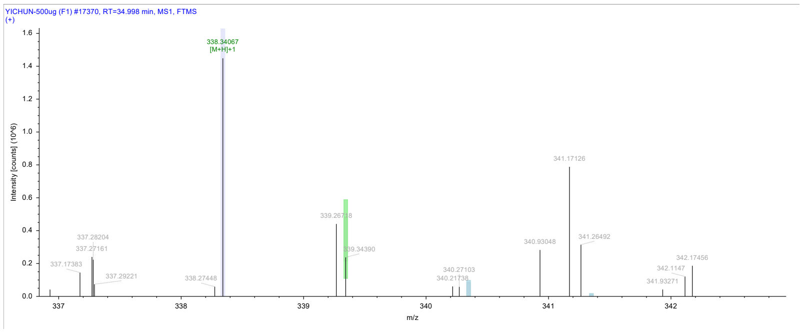

MS/MS

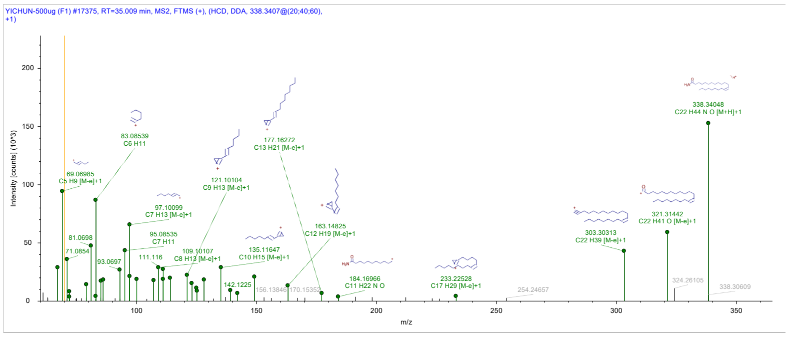

36.031 min Prenol

MS

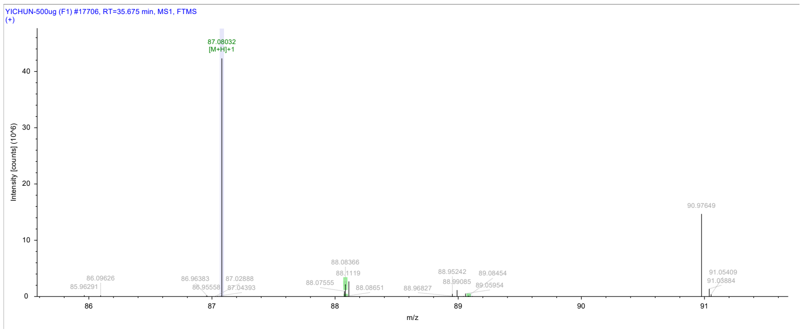

MS/MS

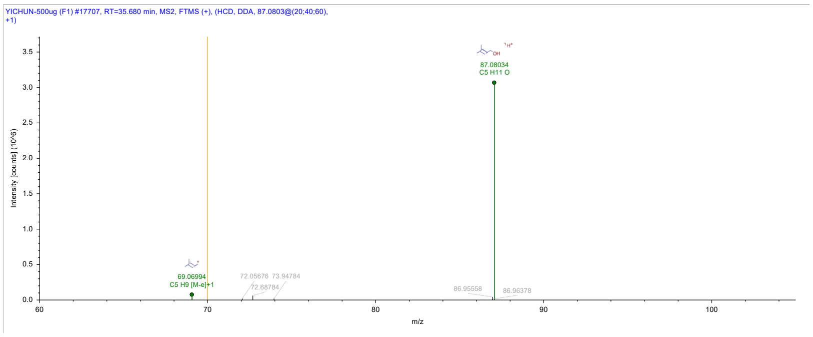

36.26 min Isothujone

MS

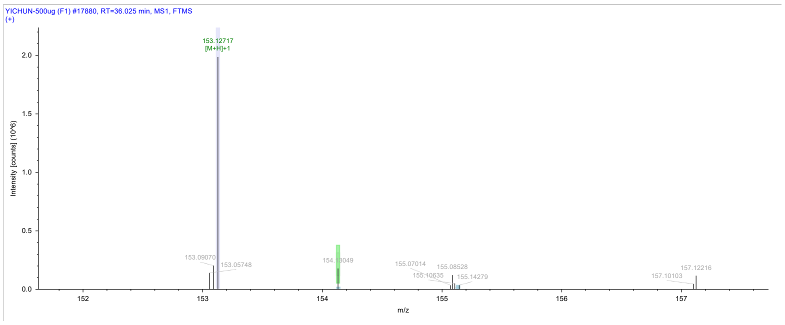

MS/MS

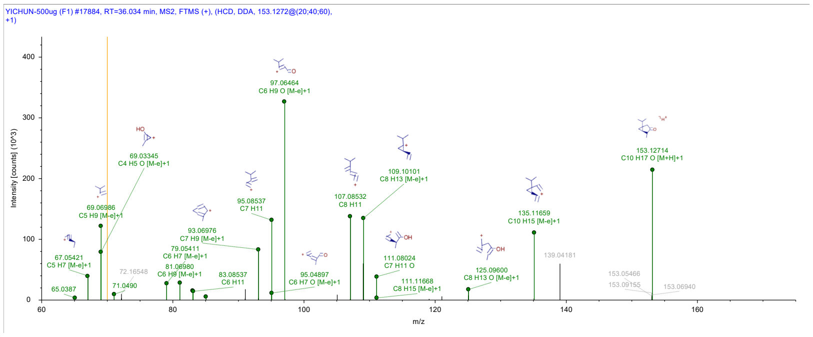

36.486 min Hexadecan-2-one

MS

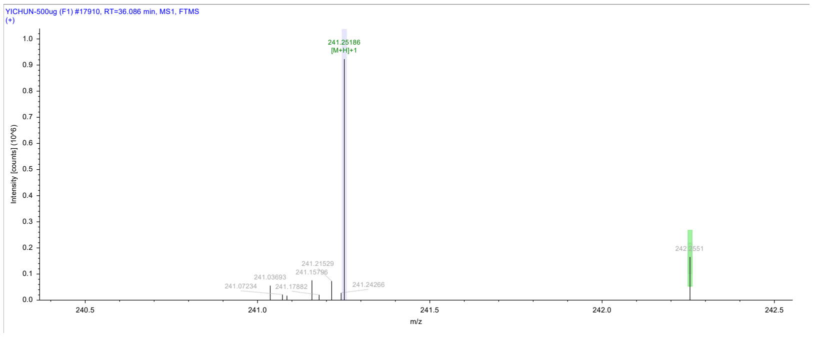

MS/MS

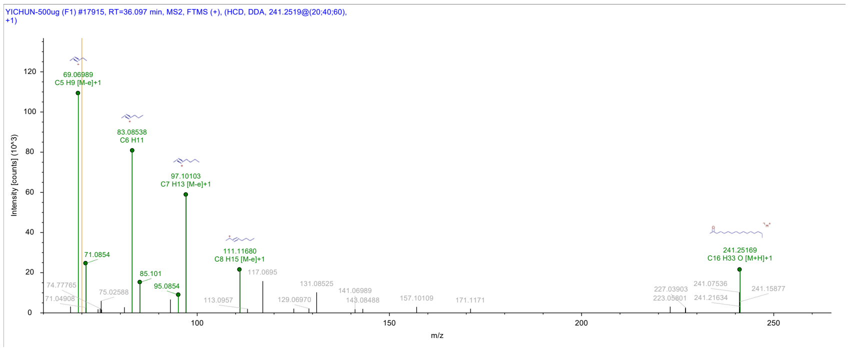

36.858 min Solwaric acid A

MS

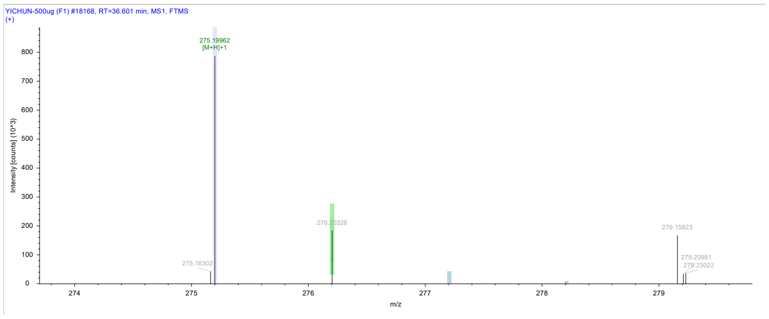

MS/MS

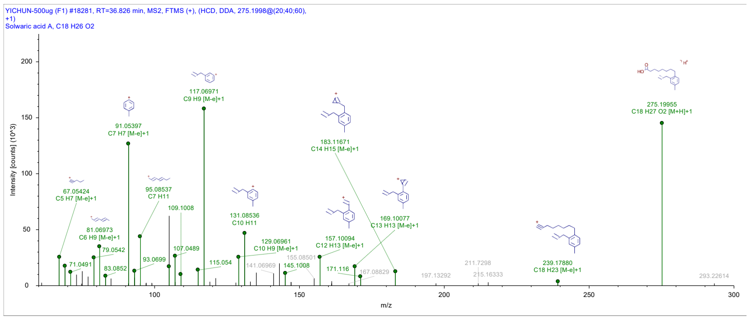

36.873 min Isoprenol

MS

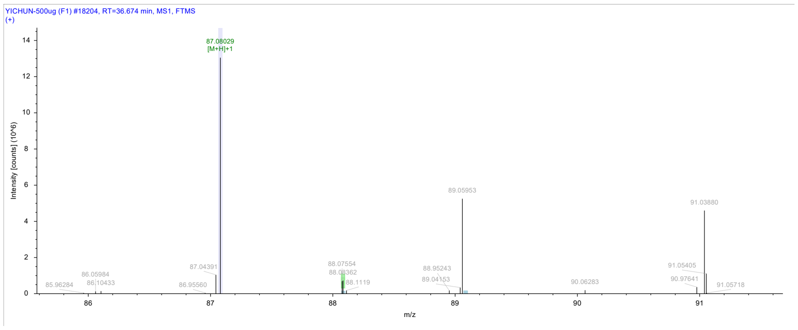

MS/MS

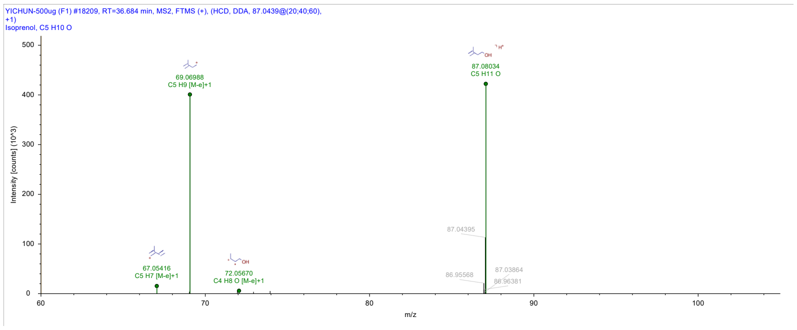

Supplement: RA-014-D4RA05643D-s001 [file RA-014-D4RA05643D-s001.pdf]
